# NMI-SO_2_Cl_2_-Mediated Amide Bond Formation: Facile Synthesis of Some Dihydrotriazolopyrimidine Amide Derivatives as Potential Anti-Inflammatory and Anti-Tubercular Agents

**DOI:** 10.3390/ph17050548

**Published:** 2024-04-24

**Authors:** Aravinda Babu, Kenchaiah Sunil, Ayyiliath Meleveetil Sajith, Eeda Koti Reddy, Sougata Santra, Grigory V. Zyryanov, Talavara Venkatesh, Somashekara Bhadrachari, Muthipeedika Nibin Joy

**Affiliations:** 1 Department of Chemistry, SSIT, Sri Siddhartha Academy of Higher Education, Tumkur 572107, Karnataka, India; aravindababu06@gmail.com (A.B.); sunilk999@gmail.com (K.S.); sajithmeleveetil@gmail.com (A.M.S.); 2 Department of Chemistry, Vignan’s Foundation for Science, Technology and Research—VFSTR (Deemed to be University), Vadlamudi, Guntur 522213, Andhra Pradesh, India; ekr.chem@gmail.com; 3 Laboratory of Organic Synthesis, Institute of Chemical Technology, Ural Federal University, 19 Mira Street, Yekaterinburg 620002, Russia; sougatasantra85@gmail.com (S.S.); g.v.zyrianov@urfu.ru (G.V.Z.); 4 I. Ya. Postovskiy Institute of Organic Synthesis, Ural Division of the Russian Academy of Sciences, 22 S. Kovalevskoy Street, Yekaterinburg 620219, Russia; 5 Department of P.G Studies and Research in Chemistry, Kuvempu University, Jnana Sahyadri, Shankaraghatta, Shimoga 577451, Karnataka, India; venkateshatalwar@gmail.com; 6 Department of Chemistry, Smt. Indira Gandhi Government First Grade Women’s College, Sagar 577401, Karnataka, India; somubchar@gmail.com

**Keywords:** dihydrotriazolopyrimidine, *N*-methylimidazole, sulfuryl chloride, amide bond formation, anti-inflammatory activity, anti-tubercular activity

## Abstract

Facile access to some novel biologically relevant dihydrotriazolopyrimidine carboxylic acid-derived amide analogues using NMI/SO_2_Cl_2_, and aromatic and aliphatic primary and secondary amines, is reported herein. The role of *N*-methylimidazole (NMI) as the base and sulfuryl chloride (SO_2_Cl_2_) as the coupling reagent has been effectively realized in accessing these molecules in good to excellent yields. The feasibility of the developed protocol has also been extended to the gram-scale synthesis of *N*-benzylbenzamide in a 75% yield from benzoic acid and benzyl amine. The newly synthesized compounds were tested via in vitro anti-inflammatory and anti-tubercular activity studies. The compounds **6aa** and **6be** were found to be the most active anti-inflammatory agents, whereas **6cb** and **6ch** were found to exhibit promising anti-tubercular potency when compared to other synthesized molecules. The structure–activity relationship (SAR) studies revealed the importance of the presence of electron-donating functionalities in enhancing the anti-inflammatory potential of the newly synthesized molecules. However, the presence of electron-withdrawing substituents was found to be significant for improving their anti-tubercular potency.

## 1. Introduction

Nitrogen-containing heterocyclic moieties play a significant role nowadays, as underlined by their presence in various pharmacologically active molecules [1,2,3]. Among the various nitrogen-containing compounds discovered so far, the heterocyclic moieties based on triazoles and dihydropyrimidines are of profound importance [4,5]. They are reported to possess various pharmacological activities, such as antimicrobial, anti-inflammatory, anticancer, antimalarial and antioxidant properties [6,7,8,9,10]. In addition to this, triazolopyrimidine derivatives exhibit acetylcholinesterase (AChE) inhibitory properties, an important factor necessary for the treatment of Alzheimer’s disease (AD) [1,2,3,11,12,13]. Among the various triazole derivatives reported hitherto, the compounds containing 1,2,4-triazole have significant applications in medicinal chemistry [14,15,16]. Hence, the synthesis of novel heterocyclic compounds containing 1,2,4-triazoles and dihydropyrimidines is highly significant in medicinal chemistry.

Inflammation is a defensive immune reaction that develops in our body against mechanical injury, pathogens or irritants. The healing process occurring in the body is initiated by inflammation after subjecting it to various physiological adaptations to minimize the tissue damage [17,18,19]. On the other hand, long-term inflammatory conditions are not very useful for the body and can lead to various disorders, like multiple sclerosis, atherosclerosis, retinitis, psoriasis, inflammatory bowel diseases, osteoarthritis and rheumatoid arthritis. Some of these inflammatory diseases can even lead to death [20,21], and hence, there is a continuous need to discover new anti-inflammatory agents from time to time. Tuberculosis (TB) is an illness caused by *Mycobacterium tuberculosis* and is considered a serious life-threatening disease worldwide [22]. TB has resulted in over 1.6 million deaths and 10.6 million clinical cases (until 2021) according to a recent report of the World Health Organization (WHO) [23]. Considering these observations, development of novel anti-TB agents has been the aim of many researchers around the globe. Nevertheless, it has been reported that only a few compounds have reached the clinical trials stage so far, which indicates a vital need to develop new anti-TB agents with excellent mechanisms of action, low toxicity, improved potency and short therapy duration profiles [24]. 

In the modern arena of drug discovery, amide and thioamide functional groups are reported to act as efficient linkers [25]. When attached to diverse heterocyclic moieties, the amides are rationalized to act as good linkers by promoting suitable binding to the active site of the protein and thereby improving its pharmacological activities [26]. Considering the biological importance of 1,2,4-triazoles, dihydropyrimidines and amides, the development of an efficient amide formation methodology to access these otherwise challenging amides is of immense potential. In view of these aforementioned observations and as a continuation of our research in developing biologically active substances [27,28,29], we were interested in synthesizing some novel dihydrotriazolopyrimidine derivatives containing amide functionality. Accordingly, we herein report the synthesis of a series of dihydrotriazolopyrimidine derivatives linked to amides by utilizing the *N*-methylimidazole (NMI) and sulfuryl chloride (SO_2_Cl_2_)-mediated amide bond formation reaction. The results of the preliminary in vitro screening of these synthesized compounds as potential anti-inflammatory and anti-tubercular agents are also reported.

## 2. Results and Discussion

### 2.1. Chemistry

**Scheme 1:**
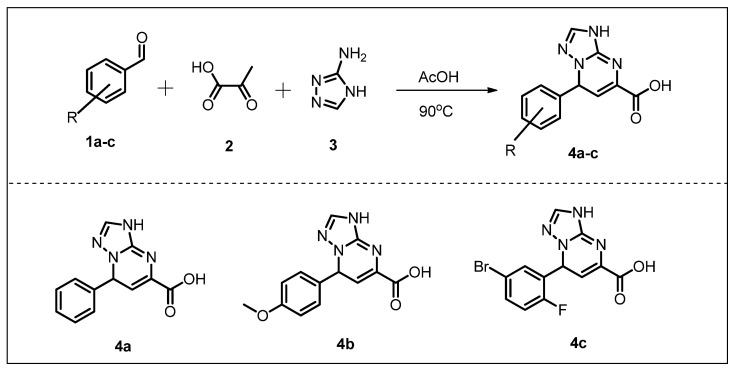
Synthesis of key acid intermediates **4a–c**.

Initially, we started our synthetic route by synthesizing the key acid intermediates **4a–c**, as summarized in Scheme 1. The one-pot, three-component reaction of different benzaldehydes **1a–c**, pyruvic acid **2** and 4*H*-1,2,4-triazol-3-amine **3** in acetic acid afforded the acid intermediates **4a–c** in good to excellent yields. The key intermediates **4a–c** were then treated with different amines **5** in view of synthesizing an array of dihydrotriazolopyrimidine derivatives containing amide functionality. 

As a model reaction for the optimization studies, we treated the acid intermediate **4a** with 4-methoxybenzylamine **5a** (Table 1). A variety of coupling reagents and bases were screened in different solvents for the reaction optimization. Gratifyingly, we obtained the expected product **6aa** in a 92% isolated yield when the reaction was carried out at room temperature by employing NMI as the base and SO_2_Cl_2_ as the coupling reagent in dichloromethane (DCM) solvent (Table 1, entry 2). The usage of other reagent combinations resulted in lesser yields of the desired product (Table 1, entries 5–9). Nevertheless, the desired product was obtained in a satisfactory yield when trifluoromethanesulfonyl chloride (TfCl) and methanesulfonyl chloride (MsCl) were employed as the coupling reagent instead of SO_2_Cl_2_ (Table 1, entries 10,11). DCM was found to be the most suitable solvent when compared to DMF and THF (Table 1, entries 12,13).

**Scheme 2:**
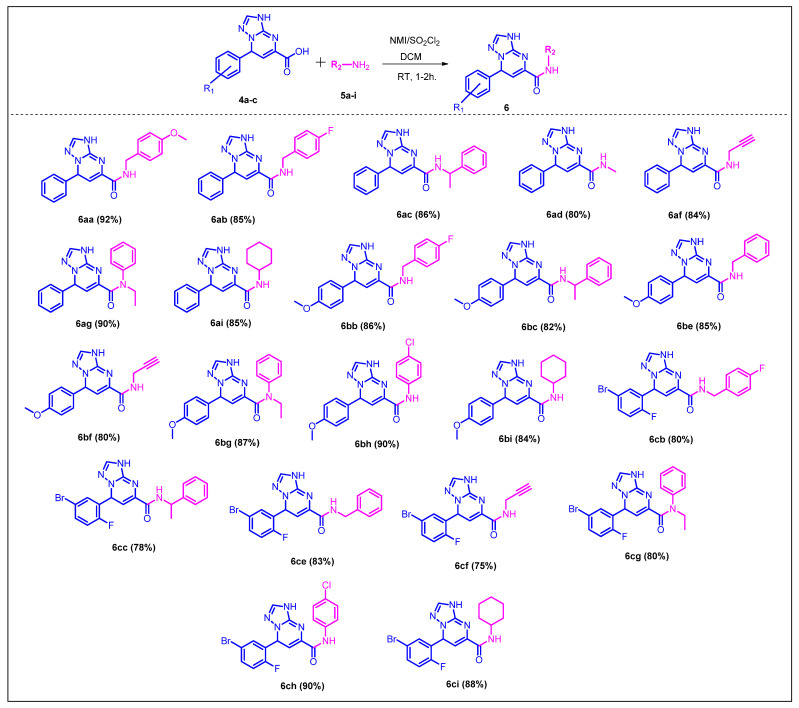
Synthesis of the final compounds; the isolated yield is given in parentheses.

After the detailed reaction optimization studies, we shifted our attention to evaluating the substrate scope of the developed methodology. Keeping this in mind, we treated the acid intermediates **4a–c** with different amines **5a–i** (Scheme 2). Gratifyingly, the amines reacted well to furnish the respective amides **6** in reasonable yields. The amines **5a**, **5g** and **5h** afforded the expected amides in excellent yields (up to a 92% isolated yield), whereas the other amines furnished the desired products in good to satisfactory yields (75–88% isolated yield).

**Scheme 3:**
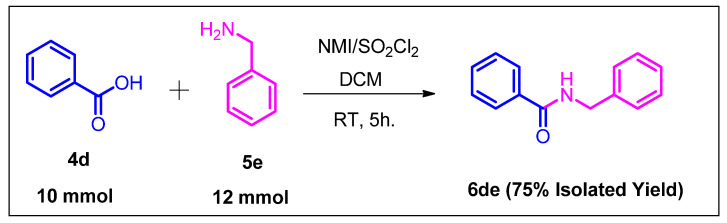
Gram-scale reaction of benzoic acid with benzyl amine.

To further expand the usefulness of the developed synthetic methodology, the protocol was further employed for scalability studies (Scheme 3). Accordingly, the gram-scale synthesis of **6de** was successfully executed using this methodology by employing benzoic acid **4d** and benzyl amine **5e** as the reactants. The method provides an alternative atom economic and cheaper protocol for accessing diverse amides. The method avoids the use of coupling agents, which need further processing to remove the side products associated with these reagents. The desired amide product **6de** was obtained in a 75% isolated yield when the reaction was carried out for 5 h.

**Scheme 4:**
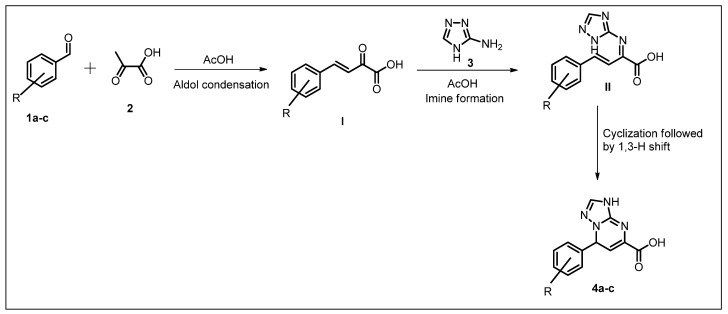
Proposed mechanism for the formation of the key acid intermediates **4a–c**.

The possible mechanism for the three-component reaction resulting in the formation of the key acid intermediates **4a–c** has been illustrated in Scheme 4 [30]. Initially, aldehydes **1a–c** and pyruvic acid **2** undergo the aldol condensation reaction to generate intermediate **I**. This intermediate undergoes the imine formation reaction with 4*H*-1,2,4-triazol-3-amine **3** in the presence of acetic acid to generate intermediate **II**. This intermediate undergoes cyclization followed by a 1,3-hydrogen shift between the nitrogen atoms to form the key acid intermediates **4a–c**.

**Scheme 5:**
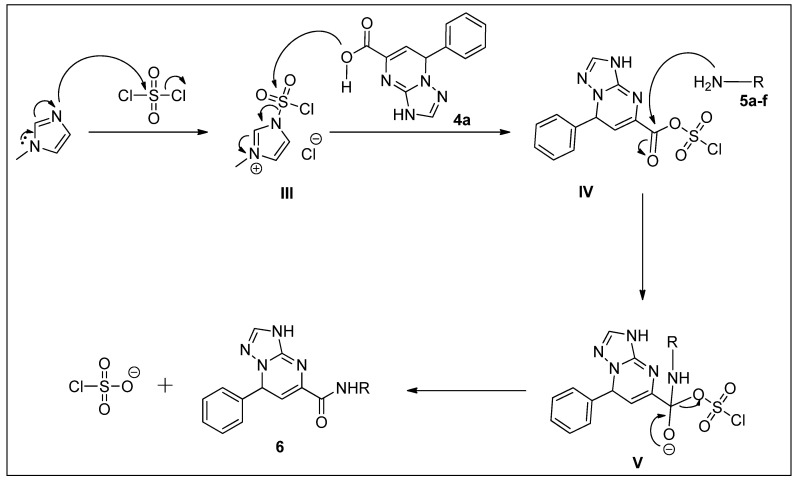
Proposed mechanism for the formation of the final products **6**.

The plausible mechanism for the formation of the different amides has been proposed in Scheme 5 by taking acid **4a** as an example [28]. During our initial optimization studies (Table 1, entry 7), we obtained the expected product in a lesser yield when NMI was replaced by triethylamine (TEA) as the base. This observation eliminated the possibility of the formation of acid chloride (by the reaction of carboxylic acid and SO_2_Cl_2_) as the first step in the reaction mechanism. Hence, it is speculated that the first step involves the generation of intermediate complex **III** by the reaction of *N*-methylimidazole and sulfuryl chloride. Subsequently, the carboxylic acid group from **4a** reacts with this complex and generates a highly activated chlorosulfonic anhydride intermediate **IV**. Finally, the nucleophilic attack of amines **5a–i** takes place on this intermediate, leading to the formation of the corresponding amides **6**.

### 2.2. Biological Activity of Synthesized Compounds

#### 2.2.1. Anti-Inflammatory Activity

All the newly synthesized dihydrotriazolopyrimidine amide derivatives **6aa–6ci** were screened for in vitro anti-inflammatory potential. The studies were performed by anti-denaturation assay and diclofenac sodium was utilized as the reference standard for the anti-inflammatory activity evaluation [31]. The compounds **6aa–6ci** and the standard were prepared in different concentrations (100, 200, 400, 800 and 1600 μg/mL) and the determination of anti-inflammatory activity was subsequently performed (Table 2).

Amongst the tested compounds, **6aa** and **6be** displayed superior anti-inflammatory activity (40 and 44% inhibition of denaturation at a 100 μg/mL concentration) when compared to the standard drug, diclofenac. The compound **6be** exhibited 44% inhibition of denaturation at a 100 μg/mL concentration, while diclofenac sodium displayed 41% inhibition at this concentration. However, the compound **6aa** showed 40% inhibition at a 100 μg/mL concentration. These two compounds also possessed a comparable percentage of inhibition at all the concentrations tested. Moreover, the compounds **6ac**, **6bc** and **6bg** also showed promising anti-inflammatory activity when compared to the reference standard. These compounds demonstrated a superior percentage of inhibition when compared with other tested compounds from the same series. In contrast, the compounds **6ab**, **6ai**, **6cb**, **6cc**, **6ce**, **6cf**, **6ch** and **6ci** showed the lowest percentage of inhibition of denaturation at all the concentrations tested. All the other tested compounds displayed moderate anti-inflammatory potential. Therefore, the compound **6be** was identified to be the most promising one among the tested compounds, as it displayed a slightly superior activity profile when compared to that of the standard drug, diclofenac sodium. 

#### 2.2.2. Anti-Tubercular Activity Studies

The in vitro anti-tubercular activity evaluation of the synthesized compounds **6aa–6ci** was carried out against various TB strains, as summarized in Table 3. The studies were carried out in two stages by the resazurin assay method by employing rifampicin and isoniazid as the reference standards [32]. Initially, the anti-tubercular activity evaluations were performed at 1, 10 and 100 μg/mL concentrations against different TB strains—namely *Mycobacterium tuberculosis* H37Rv, *Mycobacterium smegmatis* (ATCC 19420) and *Mycobacterium fortuitum* (ATCC 19542)—and the minimum inhibitory concentration (MIC) values were examined (Table 3). In the next stage, the more active compounds from the preliminary tests were subjected to a second level of screening at lower concentrations. The activity of the tested compounds against the MDR-TB strain was also evaluated during this phase, along with the three previously screened TB strains. The compounds that were active at 10 μg/mL concentrations or lower were used for this second stage of screening and the molecules that were active at 100 μg/mL or more were discarded. All the compounds selected for the second level of screening were evaluated at 0.3125, 0.625, 1.25, 2.5 and 5.0 μg/mL concentrations.

Among the newly synthesized compounds **6aa–6ci**, the anti-tubercular activity potential of some of the molecules was found to be promising, as they displayed MIC values in the range of 1 and 10 μg/mL concentrations against *Mycobacterium tuberculosis H37Rv* strain (Table 3). The compounds **6cb** and **6ch** were found to be potent at 0.625 μg/mL concentrations against the *Mycobacterium tuberculosis* H37Rv strain, whereas the compounds **6ab**, **6cc**, **6cf** and **6cg** were active at 1.25 μg/mL concentrations. Moreover, the compounds **6cb–6ci** demonstrated promising anti-tubercular activity against *Mycobacterium smegmatis* (ATCC 19420) at a concentration of 1.25 μg/mL. It is worth mentioning that most of the tested compounds were not very active against *Mycobacterium fortuitum* (ATCC 19542) when compared with the reference drug, rifampicin. Furthermore, the compounds **6cb** and **6ch** showed promising potency against the *MDR-TB* strain at 6.25 μg/mL. The other tested compounds in this series exhibited lower or modest activity profiles against the various TB strains.

#### 2.2.3. SAR Studies

**Scheme 6:**
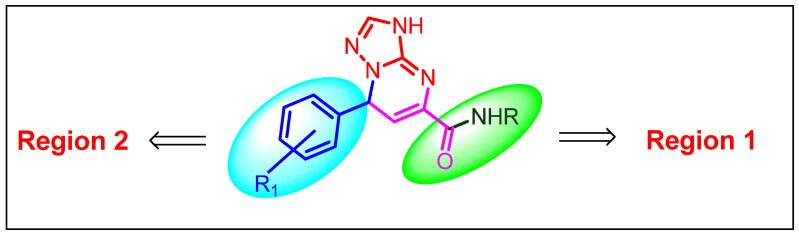
SAR studies of the newly synthesized compounds **6aa–6ci**.

After evaluating the anti-inflammatory and anti-tubercular properties of the newly synthesized dihydrotriazolopyrimidine amide derivatives **6aa–6ci**, SAR studies were carried out to determine the correlation between their promising activity profile and structural specificity (Scheme 6). In this work, we have synthesized 21 dihydrotriazolopyrimidine compounds linked to amide functionality. Our initial efforts were focused on synthesizing various types of amides (Region 1, Scheme 6) and Region 2 was restricted to three different functional groups. The compounds containing varied electronic and steric features were comprised in our anti-inflammatory and anti-tubercular activity studies. The SAR studies paved the way for realizing the importance of some specific electronic features in enhancing the overall pharmacological profile of the tested compounds.

Among the various compounds screened for the anti-inflammatory studies, **6aa** and **6be** were found to be the most active ones. The enhanced anti-inflammatory potential of **6aa** and **6be** when compared to the other tested molecules can possibly be due to the presence of electron-donating methoxy groups in these compounds. Alternatively, the compounds containing electron-withdrawing groups were not found to be active as anti-inflammatory agents. Among the compounds screened for anti-tubercular activity studies, **6cb** and **6ch** were found to be the most active ones. Moreover, the compounds **6ab**, **6cc**, **6ce**, **6cf**, **6cg** and **6ci** were also found to be active as anti-tubercular agents. In these molecules, electron-withdrawing functionalities such as F, Br or Cl are present and this could possibly be the main reason for the higher potency of these compounds. Generally, the presence of electron-withdrawing groups at the phenyl ring increases the lipophilicity of the compound, as evident from the calculated LogP and CLogP (Table 4). Presumably, this will increase their cell permeability and hence improve the overall activity of these compounds [33].

In summary, the presence of electron-donating functionalities at Regions 1 and 2 was found to be necessary for improved anti-inflammatory potential, whereas the existence of electron-withdrawing substituents (at Regions 1 and 2) significantly enhanced the anti-tubercular potential of the synthesized compounds.

## 3. Materials and Methods

### 3.1. General Considerations

All the chemicals were purchased from commercial suppliers and used as delivered. The ^1^H NMR and ^13^C NMR spectra were recorded at 400 and 100 MHz, respectively (the NMR spectra of all the new compounds are available in the Supplementary Materials). The chemical shifts were reported in parts per million (ppm) and the coupling constants in Hertz (Hz). Tetramethylsilane (TMS) (δ = 0.00 ppm) or the residual solvent peak in DMSO-*d*_6_ (δ = 2.50 ppm) and CDCl_3_ (δ = 7.26 ppm) served as the internal standard for recording [34]. The molecular weights of unknown compounds were checked by the LC-MS 6200 series supplied by Agilent Technology. The microanalyses were performed on a PerkinElmer Series II CHNS/O 2400 elemental analyzer. The melting points were determined using a Stuart SMP 3 apparatus. Thin-layer chromatography (TLC) was performed using Merck silica gel 60 F_254_ TLC plates.

### 3.2. Experimental Section

#### 3.2.1. General Procedure for the Synthesis of Acid Intermediates **4a–c**

A solution of benzaldehydes (**1a–c**) (5 mmol, 1.0 equiv.), pyruvic acid (**2**) (5 mmol, 1.0 equiv.) and 4*H*-1,2,4-triazol-3-amine (**3**) (5 mmol, 1.0 equiv.) was taken in acetic acid (10 vol) and heated at 90 °C for 1h. After the specified time, the reaction mixture was cooled to room temperature and then water (100 mL) was added to obtain a precipitate. The precipitate was filtered, again washed with water (100 mL) and the solid was dried to obtain the desired acids with different yields.

**7-Phenyl-3,7-dihydro-[1,2,4]triazolo[1,5-a]pyrimidine-5-carboxylic acid (4a)**

Yield = 82%; off-white solid. Mp 175–177 °C. ^1^H NMR (400 MHz, DMSO-*d*_6_): δ 9.93 (s, 1H, COOH), 7.62 (s, 1H, ArH), 7.34 (m, 3H, ArH), 6.26 (d, *J* = 4.0 Hz, 1H, NH), 5.79 (d, *J* = 4.0 Hz, 1H, CH). ^13^C NMR (100 MHz, DMSO-*d*_6_): δ 163.3, 150.3, 149.4, 141.3, 129.2, 128.6, 128.1, 127.1, 106.6, 59.7. LC-MS: 243.2 (M+H). Anal. Calculated for C_18_H_16_F_2_N_4_: C, 59.50; H, 4.16; N, 23.13; found: C, 59.32; H, 4.50; N, 23.11%.

**7-(4-Methoxyphenyl)-3,7-dihydro-[1,2,4]triazolo[1,5-a]pyrimidine-5-carboxylic acid (4b)**

Yield = 85%; off-white solid. Mp 171–174 °C. ^1^H NMR (400 MHz, DMSO-*d*_6_): δ 9.9 (s, 1H, COOH), 7.6 (s, 1H, ArH), 7.14 (d, *J* = 8.3 Hz, 2H, ArH), 6.91 (d, *J* = 8.3 Hz, 2H, ArH), 6.19 (d, *J* = 4.0 Hz, 1H, NH), 5.76 (d, *J* = 4.0 Hz, 1H, CH), 3.73 (s, 3H, OCH_3_). ^13^C NMR (100 MHz, DMSO-*d*_6_): δ 163.3, 159.6, 150.2, 149.1, 133.4, 128.6, 127.9, 114.5, 106.7, 59.2, 55.6. LC-MS: 273.1 (M+H). Anal. Calculated for C_18_H_16_F_2_N_4_: C, 57.35; H, 4.44; N, 20.58; found: C, 57.24; H, 4.05; N, 20.54%.

**7-(5-Bromo-2-fluorophenyl)-3,7-dihydro-[1,2,4]triazolo[1,5-a]pyrimidine-5-carboxylic acid (4c)**

Yield = 74%; pale yellow solid. Mp 210–215 °C. ^1^H NMR (400 MHz, DMSO-*d*_6_): δ 13.61 (s, 1H, COOH), 10.09 (s, 1H, ArH), 7.77–7.63 (m, 2H, ArH), 7.18 (qd, *J* = 8.2, 4.2 Hz, 1H, ArH), 6.79 (dd, *J* = 9.2, 3.1 Hz, 1H, ArH), 6.53 (d, *J* = 3.8 Hz, 1H, NH), 5.7 (d, *J* = 3.7 Hz, 1H, CH). ^13^C NMR (100 MHz, DMSO-*d*_6_): δ 163.3, 163.1, 160.8, 150.8, 149.9, 141.3 (d, *J* = 6.4 Hz), 135.6 (d, *J* = 8.1 Hz), 129.3, 117.9 (d, *J* = 22.4 Hz), 116.7 (d, *J* = 23.7 Hz), 115.8 (d, *J* = 3.0 Hz), 102.4 (d, *J* = 179.8 Hz). LC-MS: 340.1 (M+2H). Anal. Calculated for C_18_H_16_F_2_N_4_: C, 42.50; H, 2.38; N, 16.52; found: C, 42.52; H, 2.17; N, 16.45%.

#### 3.2.2. General Procedure for the Synthesis of the Final Compounds

Sulfuryl chloride (2 mmol, 2.0 equiv.) was added to a solution of N-methyl imidazole (2 mmol, 2.0 equiv.) in dichloromethane (10 vol) and stirred for 15 min. To that mixture, acids (**4a–c**) (1 mmol, 1.0 equiv.) were added, followed by amines (**5a–f**) (1.2 mmol, 1.2 equiv.). The reaction mixture was stirred for 2 h at room temperature and then quenched with water (10 vol). The organic layer was washed with saturated sodium bicarbonate solution (10 mL) and brine (10 mL). The organic layer was dried over anhydrous sodium sulfate and concentrated under a vacuum. The residue obtained was washed with dichloromethane to obtain the respective amides in varying yields.

***N*-(4-Methoxybenzyl)-7-phenyl-3,7-dihydro-[1,2,4]triazolo[1,5-a]pyrimidine-5-carboxamide (6aa)**

Yield = 92%; off-white solid. Mp 151–154 °C. ^1^H NMR (400 MHz, DMSO-*d*_6_): δ 9.7 (s, 1H, NHCH_2_), 8.85 (t, *J* = 6.2 Hz, 1H, ArH), 7.62 (s, 1H, ArH), 7.33 (dq, *J* = 14.6, 7.5 Hz, 3H, ArH), 7.21 (d, *J* = 7.9 Hz, 4H, ArH), 6.87 (d, *J* = 8.1 Hz, 2H, ArH), 6.23 (d, *J* = 3.9 Hz, 1H, NH), 5.6 (d, *J* = 4.0 Hz, 1H, CH), 4.3 (t, *J* = 4.8 Hz, 2H, CH_2_), 3.71 (s, 3H, OCH_3_). ^13^C NMR (100 MHz, DMSO-*d*_6_): δ 161.6, 158.8, 150.3, 149.4, 141.7, 131.3, 130.2, 129.3, 129.1, 128.6, 127.3, 114.1, 101.7, 59.6, 55.5, 42.6. LC-MS: 362.1 (M+H). Anal. Calculated for C_18_H_16_F_2_N_4_: C, 66.47; H, 5.30; N, 19.38; found: C, 66.36; H, 5.24; N, 19.25%.

***N*-(4-Fluorobenzyl)-7-phenyl-3,7-dihydro-[1,2,4]triazolo[1,5-a]pyrimidine-5-carboxamide (6ab)**

Yield = 86%; off-white solid. Mp 152–155 °C. ^1^H NMR (400 MHz, DMSO-*d*_6_): δ 9.87 (s, 1H, NHCH_2_), 8.95 (t, *J* = 6.0 Hz, 1H, ArH), 7.69 (m, 2H, ArH), 7.32 (dd, *J* = 8.4, 5.6 Hz 2H, ArH), 7.16 (m, 5H, ArH), 6.79 (d, *J* = 3.8 Hz, 1H, ArH), 6.52 (s, 1H, NH), 5.61 (d, *J* = 4.1 Hz, 1H, CH), 4.41 (t, *J* = 5.6 Hz, 2H, CH_2_). ^13^C NMR (100 MHz, DMSO-*d*_6_): δ 162.4, 150.2, 149.1, 133.7, 130.5, 129.4, 128.7, 115.5, 114.5, 101.9, 59, 55.6, 42.4. LC-MS: 350.1 (M+H). Anal. Calculated for C_18_H_16_F_2_N_4_: C, 65.32; H, 4.62; N, 20.05; found: C, 65.05; H, 4.25; N, 19.93%.

**7-Phenyl-*N*-(1-phenylethyl)-3,7-dihydro-[1,2,4]triazolo[1,5-a]pyrimidine-5-carboxamide (6ac)**

Yield = 86%; off-white solid. Mp 151–154 °C. ^1^H NMR (400 MHz, DMSO-*d*_6_): δ 9.65 (s, 1H, NHCH_2_), 8.72 (t, *J* = 7.4 Hz, 1H, ArH), 7.62 (d, *J* = 1.7 Hz, 1H, ArH), 7.36 (m, *J* = 6H, ArH), 7.27–7.17 (m, 3H, ArH), 6.25 (d, *J* = 3.8 Hz, 1H, NH), 5.78 (d, *J* = 4.3 Hz, 1H, CH), 5.05 (p, *J* = 7.1 Hz, 1H, CH), 1.49–1.33 (m, 3H, CH_3_). ^13^C NMR (100 MHz, DMSO-*d*_6_): δ 160.9, 150.3, 149.4, 144.6, 141.7, 130.1, 129.2, 128.7, 128.6, 127.3, 127.2, 126.5, 102.1, 59.7, 49.1, 22.4. LC-MS: 345.1 (M+H). Anal. Calculated for C_18_H_16_F_2_N_4_: C, 69.55; H, 5.54; N, 20.28; found: C, 69.55; H, 5.54; N, 20.49%.

***N*-Methyl-7-phenyl-3,7-dihydro-[1,2,4]triazolo[1,5-a]pyrimidine-5-carboxamide (6ad)**

Yield = 80%; off-white solid. Mp 145–148 °C. ^1^H NMR (400 MHz, DMSO-*d*_6_): δ 9.7 (s, 1H, NHCH_2_), 8.35 (q, *J* = 4.5 Hz, 1H, ArH), 7.62 (d, *J* = 2.4 Hz, 1H, ArH), 7.37 (dd, *J* = 8.1, 6.5 Hz, 3H, ArH), 7.24–7.2 (m, 2H, ArH), 6.23 (d, *J* = 3.9 Hz, 1H, NH), 5.6 (d, *J* = 4.0 Hz, 1H, CH), 2.69 (d, *J* = 4.5 Hz, 3H, CH_3_). ^13^C NMR (100 MHz, DMSO-*d*_6_): δ 162.8, 161.9, 160.5, 159.6, 150.1, 149.1, 135.6, 133.7, 130.1, 129.9, 129.8, 128.7, 115.6, 114.5, 101.9, 59.1, 42.4. LC-MS: 256.1 (M+H). Anal. Calculated for C_18_H_16_F_2_N_4_: C, 61.17; H, 5.13; N, 27.43; found: C, 61.43; H, 5.40; N, 27.79%.

**7-Phenyl-*N*-(prop-2-yn-1-yl)-3,7-dihydro-[1,2,4]triazolo[1,5-a]pyrimidine-5-carboxamide (6af)**

Yield = 82%; off-white solid. Mp 140–141 °C. ^1^H NMR (400 MHz, DMSO-*d*_6_): δ 9.67 (s, 1H, NHCH_2_), 8.84 (t, *J* = 5.6 Hz, 1H, ArH), 7.57 (s, 1H, ArH), 7.14 (d, *J* = 8.3 Hz, 2H, ArH), 6.9 (d, *J* = 8.3 Hz, 2H, ArH), 6.16 (d, *J* = 3.8 Hz, 1H, NH), 5.63 (d, *J* = 4.3 Hz, 1H, CH), 3.94 (dd, *J* = 5.67, 2.5 Hz, 2H, CH_2_), 3.11 (t, *J* = 2.5 Hz, 1H, CH). ^13^C NMR (100 MHz, DMSO-*d*_6_): δ 160.9, 150.3, 149.3, 144.8, 141.7, 130.1, 128.6, 127.3, 101.1, 59.7, 49.1, 22.4. LC-MS: 280.1 (M+H). Anal. Calculated for C_15_H_13_N_5_O_5_: C, 64.51; H, 4.69; N, 25.07; found: C, 64.61; H, 4.35; N, 25.04%.

***N*-Ethyl-*N*,7-diphenyl-3,7-dihydro-[1,2,4]triazolo[1,5-a]pyrimidine-5-carboxamide (6ag)**

Yield = 77%; off-white solid. Mp 168–174 °C. ^1^H NMR (400 MHz, DMSO-*d*_6_): δ 10.65 (s, 1H, NH), 7.50–7.37 (m, 6H, ArH), 7.35–7.29 (m, 4H, ArH), 6.25 (d, *J* = 3.5 Hz, 1H, ArH), 4.61 (d, *J* = 3.5 Hz, 1H), 3.93 (m, 1H, CH), 3.64 (m, 1H, CH), 1.08 (t, *J* = 7.1 Hz, 3H, CH_3_). ^13^C NMR (101 MHz, DMSO-*d*_6_): δ 163.8, 150.7, 149.3, 142.2, 135.1, 132.4, 129.5, 127.6, 117.7, 98.1, 58.9, 44.4, 12.9. LC-MS: 346.1 (M+H). Anal. Calculated for C_20_H_19_N_5_O: C, 69.55; H, 5.54; N, 20.28; found: C, 69.33; H, 5.34; N, 19.93%.

***N*-Cyclohexyl-7-phenyl-3,7-dihydro-[1,2,4]triazolo[1,5-a]pyrimidine-5-carboxamide (6ai)**

Yield = 85%; off-white solid. Mp 161–164 °C. ^1^H NMR (400 MHz, DMSO-*d*_6_): δ 10.15 (s, 1H, NH), 8.21 (d, *J* = 7.8 Hz, 1H, ArH), 7.29–719 (m, 5H, ArH), 6.55 (d, *J* = 3.6 Hz, 1H, ArH), 5.74 (d, *J* = 3.7 Hz, 1H, CH), 3.66 (m, 1H, CH), 1.84–1.65 (m, 4H, CH_2_), 1.58 (dd, *J* = 10.3 Hz, 6.5 Hz, 1H, CH), 1.26 (h, *J* = 8.2 Hz, 7.6 Hz, 4H, CH_2_), 1.15–1.03 (m, 1H, CH). ^13^C NMR (101 MHz, DMSO-*d*_6_) δ 163.4, 160.5, 150.8, 149.9, 135.6, 131.4, 117.9, 98.7, 59.7, 49, 32.9, 25.6. LC-MS: 324.2 (M+H). Anal. Calculated for C_18_H_21_N_5_O: C, 64.57; H, 6.56; N, 19.82; found: C, 64.34; H, 6.44; N, 19.74%.

***N*-(4-Fluorobenzyl)-7-(4-methoxyphenyl)-3,7-dihydro-[1,2,4]triazolo[1,5-a]pyrimidine-5-carboxamide (6bb)**

Yield = 86%; off-white solid. Mp 152–155 °C. ^1^H NMR (400 MHz, DMSO-*d*_6_): δ 9.67 (s, 1H, NHCH_2_), 8.92 (t, *J* = 6.0 Hz, 1H, ArH), 7.58 (s, 1H, ArH), 7.32 (dd, *J* = 8.4, 5.6 Hz 2H, ArH), 7.15 (m, 4H, ArH), 6.98 (m, 2H, ArH), 6.18 (d, *J* = 3.8 Hz, 1H, NH), 5.65 (d, *J* = 4.1 Hz, 1H, CH), 4.41 (t, *J* = 5.6 Hz, 2H, CH_2_), 3.73 (s, 3H, OCH_3_). ^13^C NMR (100 MHz, DMSO-*d*_6_): δ 162.4, 150.2, 149.1, 133.7, 130.5, 129.4, 128.7, 115.5, 114.5, 101.9, 59, 55.6, 42.4. LC-MS: 380.1 (M+H). Anal. Calculated for C_18_H_16_F_2_N_4_: C, 63.32; H, 4.78; N, 18.46; found: C, 63.68; H, 5.06; N, 18.62%.

**7-(4-Methoxyphenyl)-*N*-(1-phenylethyl)-3,7-dihydro-[1,2,4]triazolo[1,5-a]pyrimidine-5-carboxamide (6bc)**

Yield = 82%; off-white solid. Mp 162–165 °C. ^1^H NMR (400 MHz, DMSO-*d*_6_): δ 9.59 (s, 1H, NHCH_2_), 8.72 (t, *J* = 7.4 Hz, 1H, ArH), 7.6 (s, 1H, ArH), 7.32 (d, *J* = 8.3 Hz 2H, ArH), 7.27–7.1 (m, 4H, ArH), 6.93 (t, *J* = 6.6 Hz 2H, ArH), 6.19 (d, *J* = 3.8 Hz, 1H, NH), 5.78 (d, *J* = 4.3 Hz, 1H, CH), 5.05 (p, *J* = 7.3 Hz, 1H, CH), 3.79 (s, 3H, OCH_3_), 1.49–1.33 (m, 3H, CH_3_). ^13^C NMR (100 MHz, DMSO-*d*_6_): δ 160.9, 159.6, 149.9, 149.1, 144.7, 133.7, 129.8, 128.7, 127.2, 126.5, 114.5, 102.3, 59.1, 55.6, 49.1, 22.3. LC-MS: 376.1 (M+H). Anal. Calculated for C_18_H_16_F_2_N_4_: C, 67.18; H, 5.64; N, 18.65; found: C, 67.07; H, 5.54; N, 18.45%.

***N*-Benzyl-7-(4-methoxyphenyl)-3,7-dihydro-[1,2,4]triazolo[1,5-a]pyrimidine-5-carboxamide (6be)**

Yield = 85%; off-white solid. Mp 157–160 °C. ^1^H NMR (400 MHz, DMSO-*d*_6_): δ 9.7 (s, 1H, NHCH_2_), 8.94 (t, *J* = 6.0 Hz, 1H, ArH), 7.6 (s, 1H, ArH), 7.28 (m, 5H, ArH), 7.17 (d, *J* = 8.2 Hz, 2H, ArH), 6.92 (d, *J* = 8.2 Hz, 2H, ArH), 6.19 (d, *J* = 3.8 Hz, 1H, NH), 5.69 (d, *J* = 4.1 Hz, 1H, CH), 4.39 (t, *J* = 5.6 Hz, 2H, CH_2_), 3.73 (s, 3H, OCH_3_). ^13^C NMR (100 MHz, DMSO-*d*_6_): δ 161.9, 159.6, 150.2, 149.1, 139.4, 133.7, 130.1, 128.7, 127.9, 127.4, 114.5, 101.9, 59.1, 55.6, 43.1. LC-MS: 362.1 (M+H). Anal. Calculated for C_18_H_16_F_2_N_4_: C, 66.47; H, 5.30; N, 19.38; found: C, 66.68; H, 5.30; N, 19.36%.

**7-(4-Methoxyphenyl)-*N*-(prop-2-yn-1-yl)-3,7-dihydro-[1,2,4]triazolo[1,5-a]pyrimidine-5-carboxamide (6bf)**

Yield = 90%; off-white solid. Mp 141–144 °C. ^1^H NMR (400 MHz, DMSO-*d*_6_): δ 9.67 (s, 1H, NHCH_2_), 8.84 (t, *J* = 5.6 Hz, 1H, ArH), 7.57 (s, 1H, ArH), 7.14 (d, *J* = 8.3 Hz, 2H, ArH), 6.9 (d, *J* = 8.3 Hz, 2H, ArH), 6.16 (d, *J* = 3.8 Hz, 1H, NH), 5.63 (d, *J* = 4.3 Hz, 1H, CH), 3.94 (dd, *J* = 5.67, 2.5 Hz, 2H, CH_2_), 3.71 (s, 3H, OCH_3_), 3.11 (t, *J* = 2.5 Hz, 1H, CH). ^13^C NMR (100 MHz, DMSO-*d*_6_): δ 167.7, 159.6, 150.2, 149.1, 133.7, 129.7, 128.7, 114.5, 102.3, 73.7, 58.9, 55.6. LC-MS: 310.1 (M+H). Anal. Calculated for C_18_H_16_F_2_N_4_: C, 62.13; H, 4.89; N, 22.64; found: C, 62.36; H, 4.59; N, 22.97%.

***N*-Ethyl-7-(4-methoxyphenyl)-*N*-phenyl-3,7-dihydro-[1,2,4]triazolo[1,5-a]pyrimidine-5-carboxamide (6bg)**

Yield = 76%; off-white solid. Mp 163–166 °C. ^1^H NMR (400 MHz, DMSO-d_6_): δ 10.65 (s, 1H, NH), 7.50–7.37 (m, 3H, ArH), 7.35–7.29 (m, 2H, ArH), 6.78–6.69 (m, 2H, ArH), 6.60 (d, *J* = 8.2 Hz, 2H, ArH), 6.25 (d, *J* = 3.5 Hz, 1H, ArH), 4.61 (d, *J* = 3.5 Hz, 1H), 3.93 (m, 1H, CH), 3.73 (s, 3H, OCH_3_), 3.64 (m, 1H, CH), 1.08 (t, *J* = 7.1 Hz, 3H, CH_3_). ^13^C NMR (101 MHz, DMSO-*d*_6_): δ 163.9, 159.9, 149.5, 142.4, 131.9 130.1, 129.2, 127.9, 114.4, 100.5, 58.1, 55.7, 44.6, 12.9. LC-MS: 375.1 (M+H). Anal. Calculated for C_19_H_23_N_5_O_2_: C, 67.18; H, 5.64; N, 18.65; found: C, 66.98; H, 5.60; N, 18.85%.

***N*-(4-Chlorophenyl)-7-(4-methoxyphenyl)-3,7-dihydro-[1,2,4]triazolo[1,5-a]pyrimidine-5-carboxamide (6bh)**

Yield = 81%; off-white solid. Mp 152–155 °C. ^1^H NMR (401 MHz, DMSO-*d*_6_) δ 10.42 (s, 1H, NH), 7.66–7.71 (m, 1H, ArH), 7.44–7.4 (m, 1H, 4H), 7.33 (m, 2H, ArH), 7.01 (m, 2H, ArH), 6.33 (dd, *J* = 9.1, 3.1 Hz, 1H), 5.93 (t, *J* = 3.5 Hz, 1H). ^13^C NMR (101 MHz, DMSO-*d*_6_) 6 160.91, 160.43, 150.85, 149.81, 135.62 (d, *J* = 8.1 Hz), 131.62, 129.02, 128.24, 122.53, 100.47, 60.01 (d, *J* = 41.3 Hz). LC-MS: 382.1 (M+H). Anal. Calculated for C_19_H_16_ClN_5_O_2_: C, 59.77; H, 4.22; N, 18.34; found: C, 59.62; H, 4.53; N, 18.52%.

***N*-Cyclohexyl-7-(4-methoxyphenyl)-3,7-dihydro-[1,2,4]triazolo[1,5-a]pyrimidine-5-carboxamide (6bi)**

Yield = 80%; off-white solid. Mp 162–165 °C. ^1^H NMR (400 MHz, DMSO-*d*_6_): δ 10.15 (s, 1H, NH), 8.21 (d, *J* = 7.8 Hz, 1H, ArH), 7.29–719 (m, 2H, ArH), 7.03–6.92 (m, 2H, ArH), 6.55 (d, *J* = 3.6 Hz, 1H, ArH), 5.74 (d, *J* = 3.7 Hz, 1H, CH), 3.76 (s, 3H, OCH_3_), 3.66 (m, 1H, CH), 1.84–1.65 (m, 4H, CH_2_), 1.58 (dd, *J* = 10.3 Hz, 6.5 Hz, 1H, CH), 1.26 (h, *J* = 8.2 Hz, 7.6 Hz, 4H, CH_2_), 1.15–1.03 (m, 1H, CH). ^13^C NMR (100 MHz, DMSO-*d*_6_): δ 160.4, 160, 150.6, 132.6, 130.2, 129, 114.8, 101.8, 58.4, 55.7, 49, 32.6, 25 LC-MS: 354.4 (M+H). Anal. Calculated for C_19_H_23_N_5_O_2_: C, 64.57; H, 6.56; N, 19.82; found: C, 64.34; H, 6.44; N, 19.74%.

**7-(5-Bromo-2-fluorophenyl)-*N*-(4-fluorobenzyl)-3,7-dihydro-[1,2,4]triazolo[1,5-a]pyrimidine-5-carboxamide (6cb)**

Yield = 80%; off-white solid. Mp 176–178 °C. ^1^H NMR (400 MHz, DMSO-*d*_6_): δ 9.92 (s, 1H, NHCH_2_), 8.97 (t, *J* = 5.9 Hz, 1H, ArH), 7.77–7.63 (m, 2H, ArH), 7.32 (dd, *J* = 8.4, 5.5 Hz, 2H, ArH), 7.23–7.08 (m, 3H, ArH), 6.86–6.74 (m, 1H, ArH), 6.53 (d, *J* = 3.6 Hz, 1H, ArH), 5.63 (d, *J* = 3.6 Hz, 1H, NH), 4.43–4.27 (t, *J* = 4.8 Hz, 2H, CH_2_). ^13^C NMR (100 MHz, DMSO-*d*_6_): δ 163.3, 162.9, 161.6, 160.8, 160.5, 150.7, 149.9, 141.7, 135.5, 131.3, 129.9, 117.9, 116.6, 115.8, 115.4, 98.8, 59.7, 42.4. LC-MS: 446.1 (M+H). Anal. Calculated for C_18_H_16_F_2_N_4_: C, 51.14; H, 3.16; N, 15.69; found: C, 51.28; H, 2.99; N, 15.31%.

**7-(5-Bromo-2-fluorophenyl)-*N*-(1-phenylethyl)-3,7-dihydro-[1,2,4]triazolo[1,5-a]pyrimidin-5-carboxamide (6cc)**

Yield = 78%; off-white solid. Mp 172–174 °C. ^1^H NMR (400 MHz, DMSO-*d*_6_): δ 9.93 (s, 1H, NHCH_2_), 8.74 (t, *J* = 7.1 Hz, 1H, ArH), 7.72 (m, 1H, ArH), 7.68 (d, *J* = 2.3 Hz, 2H, ArH), 7.32 (dd, *J* = 6.6, 4.4 Hz, 5H, ArH), 7.20 (m, 2H, ArH), 6.89–6.77 (m, 1H, ArH), 6.53 (d, *J* = 3.6 Hz, 1H, ArH),1.42 (dd, *J* = 7.0, 4.7 Hz, 3H, CH_3_). ^13^C NMR (100 MHz, DMSO-*d*_6_): δ 163.3, 160.9, 160.7, 150.7, 149.8, 144.6, 141.8, 135.6, 131.3, 128.7, 127.2, 126.5, 117.9, 116.7, 115.9, 98.9, 49.1, 22.3. LC-MS: 442.1 (M+H). Anal. Calculated for C_18_H_16_F_2_N_4_: C, 54.31; H, 3.87; N, 15.83; found: C, 54.49; H, 3.50; N, 16.03%.

***N*-Benzyl-7-(5-bromo-2-fluorophenyl)-3,7-dihydro-[1,2,4]triazolo[1,5-a]pyrimidine-5-carboxamide (6ce)**

Yield = 83%; off-white solid. Mp 171–174 °C. ^1^H NMR (400 MHz, DMSO-*d*_6_): δ 9.93 (s, 1H, NHCH_2_), 8.98 (t, *J* = 5.9 Hz, 1H, ArH), 7.74–7.61 (m, 2H, ArH), 7.34–7.12 (m, 6H, ArH), 6.79 (dd, *J* = 9.3, 3.1 Hz, 1H, ArH), 6.52 (d, *J* = 3.6 Hz, 1H, ArH), 5.64 (d, *J* = 3.6 Hz, 1H, NH), 4.43–4.27 (t, *J* = 4.8 Hz, 2H, CH_2_). ^13^C NMR (100 MHz, DMSO-*d*_6_): δ 163.3, 161.5, 160.9, 150.8, 149.9, 141.8, 139.3, 135.6, 131.3, 129.1, 128.7, 127.9, 127.3, 118, 117.8, 116.8, 116.5, 115.8, 59.7, 43.1. LC-MS: 428.1 (M+H). Anal. Calculated for C_18_H_16_F_2_N_4_: C, 53.29; H, 3.53; N, 16.35; found: C, 53.22; H, 3.75; N, 16.60%.

**7-(5-Bromo-2-fluorophenyl)-*N*-(prop-2-yn-1-yl)-3,7-dihydro-[1,2,4]triazolo[1,5-a]pyrimidine-5-carboxamide (6cf)**

Yield = 75%; off-white solid. Mp 162–164 °C. ^1^H NMR (400 MHz, DMSO-*d*_6_): δ 9.88 (s, 1H, NHCH_2_), 8.88 (t, *J* = 5.6 Hz, 1H, ArH), 7.73 (m, 1H, ArH), 7.68 (s, 1H, ArH), 7.21 (m, 1H, ArH), 6.79 (d, *J* = 4.3 Hz, 1H, CH), 6.51 (d, *J* = 3.8 Hz, 1H, NH), 5.61 (d, *J* = 4.3 Hz, 1H, CH), 3.94 (dd, *J* = 5.67, 2.5 Hz, 2H, CH_2_). ^13^C NMR (100 MHz, DMSO-*d*_6_): δ 167.7, 159.6, 150.2, 149.1, 133.7, 129.7, 128.7, 114.5, 102.3, 58.9, 55.6. LC-MS: 376.1 (M+H). Anal. Calculated for C_18_H_16_F_2_N_4_: C, 47.89; H, 2.95; N, 18.62; found: C, 47.79; H, 2.66; N, 18.25%.

**7-(5-Bromo-2-fluorophenyl)-*N*-ethyl-*N*-phenyl-3,7-dihydro-[1,2,4]triazolo[1,5-a]pyrimidine-5-carboxamide (6cg)**

Yield = 77%; off-white solid. Mp 177–181 °C. ^1^H NMR (400 MHz, DMSO-*d*_6_) δ 10.27 (s, 1H, NH), 7.67 -7.52 (m, 2H, ArH), 7.28–7.18 (m, 4H, ArH), 7.18–7.1 (me, ZF), 6,2 (dd, *J* = 3.7, 1.3 Hz, 1H, ArH), 5.87 (s, 1H, CH), 4.61 (d, *J* = 3.5 Hz, 1H, CH), 3.86 (m, 1H, CH_2_CH_3_), 3.63 (m, 1H, CH_2_CH_3_), 1.05 (t, *J* = 7.1 Hz, 3H, CH_3_). ^13^C NMR (101 MHz, DMSO-*d*_6_): δ 163.8, 150.7, 149.3, 142.2, 135.1, 132.4, 129.5, 127.6, 117.7, 98.1, 58.9, 44.4, 12.9. LC-MS: 443.1 (M+2H). Anal. Calculated for C_18_H_19_BrFN_5_O: C, 51.44; H, 4.56; N, 16.66; found: C, 51.23; H, 4.68; N, 16.77%.

**7-(5-Bromo-2-fluorophenyl)-*N*-(4-chlorophenyl)-3,7-dihydro-[1,2,4]triazolo[1,5-a]pyrimidine-5-carboxamide (6ch)**

Yield = 74%; yellow solid. Mp 144–147 °C. 1H NMR (401 MHz, DMSO-*d*_6_) δ 10.53 (dd, *J* = 8.1, 3.2 Hz, 1H,), 10.16 (s, 1H, NH), 7.83–7.67 (m, 4H, ArH), 7.44–7.36 (m, 2H, ArH), 7.22 (m, 1H, ArH), 6.91 (dd, *J* = 9.1, 3.1 Hz, 1H), 6.60 (d, *J* = 3.7 Hz, 1H), 5.90 (t, *J* = 3.5 Hz, 1H). 13C NMR (101 MHz, DMSO-*d*_6_): δ 160.9, 160.4, 150.8, 149.8, 135.6, 131.6, 129.1, 128.2, 122.5, 100.5, 60.1. LC-MS: 449.1 (M+2H). Anal. Calculated for C_18_H_19_BrFN_5_O: C, 48.18; H, 2.70; N, 15.61; found: C, 48.13; H, 2.92; N, 15.72%.

**7-(5-Bromo-2-fluorophenyl)-*N*-cyclohexyl-3,7-dihydro-[1,2,4]triazolo[1,5-a]pyrimidine-5-carboxamide (6ci)**

Yield = 78%; off-white solid. Mp 173–177 °C. ^1^H NMR (400 MHz, DMSO-*d*_6_) δ 9.80 (s, 1H, NH), 8.15 (d, *J* = 7.7 Hz, 1H, ArH), 7.73 (dd, *J* = 8.8, 53.2 Hz, 1H, ArH), 7.69 (s, 1H, ArH), 7.20 (m, 1H, ArH), 6.80 (dd, *J* = 9.4, 3.1 Hz, 1H, ArH), 6.52 (d, *J* = 3.7 Hz, 1H, ArH), 5.62 (d, *J* = 3.7 Hz, 1H, CH), 3.65 (m, 1H, CH_2_), 1.85–1.66 (m, 4H, CH_2_), 1.66–1.52 (m, 1H, CH), 1.34–1.17 (m, 4H, CH_2_), 1.47–1.01 (m, 1H, CH). ^13^C NMR (101 MHz, DMSO-*d*_6_): δ 163.4, 160.5, 150.8, 149.9, 141.9, 135.6, 131.4, 117.9, 116.7, 115.9, 98.7, 59.7, 49, 32.9, 25.6, 25.3. LC-MS: 421.1 (M+2H). Anal. Calculated for C_18_H_19_BrFN_5_O: C, 51.44; H, 4.56; N, 16.66; found: C, 51.23; H, 4.68; N, 16.77%.

#### 3.2.3. General Procedure for the Gram-Scale Reaction

Sulfuryl chloride (20 mmol, 2.0 equiv.) was added to a solution of N-methyl imidazole (20 mmol, 2.0 equiv.) in dichloromethane (10 vol) and stirred for 15 min. To that mixture, benzoic acid **4d** (10 mmol, 1.0 equiv.) was added, followed by benzyl amine **5e** (1.2 equiv.). The reaction mixture was stirred for 5 h at room temperature and then quenched with water (10 vol). The organic layer was washed with saturated sodium bicarbonate solution (10 mL) and brine (10 mL). The organic layer was dried over anhydrous sodium sulfate and concentrated under a vacuum. The residue obtained was washed with dichloromethane to obtain the desired *N*-benzyl-benzamide as a white solid in a 75% yield. The melting point and spectral characteristics were found to match with the reported values [35].

#### 3.2.4. Procedure for Determining In Vitro Anti-Inflammatory Activity: Anti-Denaturation Assay

The experiment was performed according to a previously reported protocol [36]. The extracts of the target compounds or drugs were dissolved in a minimum quantity of DMSO and diluted with phosphate buffer (0.2 M, PH 7.4). It was carefully noted that the final concentration of DMSO in all the solutions was less than 2.5%. The test solution (4 mL) containing various concentrations of the target compounds was mixed with 1 mL of 1 mM solution of albumin in phosphate buffer and incubated for 15 min at 37 °C. Denaturation was induced by placing the reaction mixture in a water bath at 70 °C for 15 min. The reaction mixture was cooled after 15 min, and the turbidity was measured at 660 nm. A control experiment was also carried out without adding the tested target compounds. Diclofenac sodium was employed as the standard drug for reference purposes. The percentage of the inhibition of denaturation was calculated from the control by using the following formula:

% of Inhibition = 100 × (At − Ac)/At

where At = optical density of the test solution; Ac = optical density of the control.

#### 3.2.5. Procedure for Determining Anti-Tubercular Potential

The in vitro antimycobacterial activity of the target compounds were determined by the resazurin assay method. The compounds were examined against *M. tuberculosis* H37Rv American Type Culture Collection (ATCC) 27294 and non-tubercular mycobacterial (NTM) species such as the *M. smegmatis* (MC2) ATCC 19420, *M. fortuitum* ATCC 19542 and MDR-TB strains. The MIC values for each target compound were determined against the tested tubercular strains. The standard drugs used for reference were isoniazid and rifampicin. The *M. tuberculosis* strains were full-grown in Middlebrook 7H9 broth (Difco BBL, Sparks, MD, USA) and supplemented by 10% oleic albumin dextrose catalase (OADC, Becton Dickinson, Sparks, MD, USA). Using the same medium, the culture was then diluted to McFarland 2 standard. A total of 50 mL of the culture from this standard solution was then added to 150 mL of fresh medium in 96 well microtiter plates. The test compounds were prepared as stock solutions (2 mg/mL) in N,N-Dimethyl formamide (DMF). Initially, the target compounds were tested at 1, 10 and 100 μg/mL concentrations. Later, the second level of testing was carried out for the more active compounds at 0.3125, 0.625, 1.25, 2.5 and 5 μg/mL concentrations. The control tubes were made up to the same volumes of DMF without any substrate. After incubating the stock solution at 37 °C for 7 days, each tube had 20 mL of 0.01% resazurin in water (Sigma, St. Louis, MO, USA) added to it. Resazurin is a redox dye that is blue in the oxidized state and turns pink when reduced by the growth of viable cells. The control tubes showed a change of color from blue to pink after 1 h at 37 °C. The test compounds that prevented the color change of the dye were considered to be inhibitory against the tested TB strains. Each experiment was carried out in triplicate.

## 4. Conclusions

We have successfully synthesized a series of novel dihydrotriazolopyrimidine amides by utilizing NMI-SO_2_Cl_2_-mediated amide bond formation reactions. The developed protocol can be employed as an alternative methodology to access challenging heterocyclic amide bonds with multiple hetero atoms. As evident from the success of our gram-scale reaction between benzoic acid and benzyl amine, this protocol could be applied to the coupling reaction of simple molecules. All the newly synthesized molecules were screened for their in vitro anti-inflammatory and anti-tubercular potential, and from those studies, it was found that some of the compounds exhibited promising activity profiles when compared with their respective reference standards. The SAR studies underlined the importance of the presence of electron-donating substituents in improving the anti-inflammatory potential. However, the presence of electron-withdrawing functionalities was found to be necessary for enhancing the anti-tubercular activity of the synthesized compounds.

## Figures and Tables

**Table 1:** Optimization of the reaction conditions ^a^.

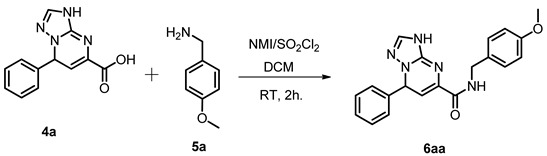					
Entry	Acid (Equiv.)	Amine (Equiv.)	NMI (Equiv.)	SO_2_Cl_2_ (Equiv.)	Yield ^b^ 6aa (%)
1	1	1	2	2	80
**2 ^c^**	**1**	**1.2**	**2**	**2**	**92**
3	1.2	1	2	2	80
4	1	1.2	1	1	75
Deviation from the above standard conditions (entry 2)					
5	EDC HCl and HOBT instead of NMI-SO_2_Cl_2_				65
6	DIPEA and HATU instead of NMI- SO_2_Cl_2_				70
7	TEA instead of NMI				40
8	T3P instead of SO_2_Cl_2_				50
9	TCFH instead of SO_2_Cl_2_				75
10	TfCl instead of SO_2_Cl_2_				82
11	MsCl instead of SO_2_Cl_2_				80
12	DMF instead of DCM				70
13	THF instead of DCM				60

^a^ Reaction conditions: acid **4a** (1.0 mmol), amine **5a** (1.2 mmol), NMI (2.0 mmol), SO_2_Cl_2_ (2.0 mmol), solvent (3 mL), RT, 2 h. ^b^ Isolated yield. ^c^ Optimized reaction condition in bold.

**Table 2:** In vitro anti-inflammatory activity studies of compounds **6aa–6ci** ^a^.

Compounds	% Inhibition of Denaturation at Different Concentrations				
	100 μg/mL	200 μg/mL	400 μg/mL	800 μg/mL	1600 μg/mL
**6aa**	40 ± 0.65	52 ± 0.79	62 ± 1.45	74 ± 0.59	88 ± 0.38
**6ab**	20 ± 0.19	32 ± 0.23	45 ± 0.36	58 ± 0.85	70 ± 1.14
**6ac**	37 ± 0.49	49 ± 0.54	60 ± 1.10	76 ± 1.04	84 ± 0.85
**6ad**	22 ± 0.49	33 ± 1.23	44 ± 0.47	60 ± 0.67	72 ± 0.84
**6af**	25 ± 0.27	38 ± 0.28	49 ± 1.01	62 ± 0.08	75 ± 0.66
**6ag**	18 ± 0.21	29 ± 0.68	40 ± 0.35	52 ± 0.58	65 ± 0.93
**6ai**	8 ± 0.45	20 ± 0.45	33 ± 1.12	46 ± 0.16	57 ± 0.17
**6bb**	21 ± 0.47	32 ± 0.54	44 ± 0.44	59 ± 0.55	72 ± 1.06
**6bc**	35 ± 1.22	48 ± 1.06	60 ± 1.04	71 ± 0.63	82 ± 1.08
**6be**	44 ± 0.49	55 ± 0.18	67 ± 0.76	80 ± 0.34	94 ± 0.48
**6bf**	24 ± 1.22	39 ± 0.75	50 ± 0.85	61 ± 1.12	70 ± 1.42
**6bg**	36 ± 0.76	47 ± 1.12	59 ± 0.45	70 ± 0.26	84 ± 1.14
**6bh**	23 ± 0.74	32 ± 0.58	40 ± 0.77	53 ± 1.02	69 ± 0.92
**6bi**	17 ± 0.18	27 ± 0.85	39 ± 0.64	50 ± 1.15	61 ± 1.36
**6cb**	8 ± 0.46	19 ± 0.18	30 ± 0.05	43 ± 1.03	55 ± 0.05
**6cc**	6 ± 1.05	17 ± 1.16	26 ± 0.65	38 ± 0.12	50 ± 0.45
**6ce**	10 ± 1.11	25 ± 1.12	35 ± 0.14	47 ± 0.04	59 ± 0.12
**6cf**	11 ± 1.05	23 ± 0.17	36 ± 0.65	48 ± 0.02	56 ± 1.01
**6cg**	20 ± 0.67	33 ± 0.69	45 ± 0.88	58 ± 0.47	70 ± 0.30
**6ch**	14 ± 0.14	24 ± 0.44	36 ± 1.09	50 ± 0.51	63± 0.14
**6ci**	12 ± 0.56	25 ± 0.15	38 ± 1.54	51 ± 1.14	60 ± 0.17
Diclofenac	41 ± 0.16	57 ± 0.48	80 ± 0.84	86 ± 1.02	89 ± 1.06

^a^ The experiments were performed in triplicate and expressed as the mean ± SD.

**Table 3:** Anti-tubercular activity data of the synthesized compounds **6aa–6ci**.

Preliminary In Vitro Screening Results, MIC (μg/mL)					Second-Level Screening Results, MIC (μg/mL)			
Compound	MTB ^a^	MS ^b^	MF ^c^	% ^d^	MTB	MS	MF	MDR-TB
**6aa**	>100	>100	>100	0	-	-	-	-
**6ab**	1 ± 0.20	10 ± 0.14	10 ± 0.35	90	1.25 ± 0.38	2.5 ± 0.16	>5	12.5 ± 0.28
**6ac**	>100	>100	>100	0	-	-	-	-
**6ad**	10 ± 0.14	10 ± 0.23	>100	<90	>5	>5	>5	>50
**6af**	10 ± 0.28	10 ± 0.22	10 ± 0.12	<90	>5	>5	>5	>50
**6ag**	>100	>100	>100	0	-	-	-	-
**6ai**	>100	>100	>100	0	-	-	-	-
**6bb**	10 ± 0.45	10 ± 0.80	>100	<90	>5	-	>5	>50
**6bc**	>100	>100	>100	0	-	-	-	-
**6be**	>100	>100	>100	0	-	-	-	-
**6bf**	>100	>100	>100	0	-	-	-	-
**6bg**	>100	>100	>100	0	-	-	-	-
**6bh**	10 ± 0.32	10 ± 0.04	10 ± 0.28	10± 0.30	10 ± 0.45	10 ± 0.36	10 ± 0.20	10 ± 0.55
**6bi**	>100	>100	>100	0	-	-	-	-
**6cb**	1 ± 0.18	1 ± 0.29	10 ± 0.45	95	0.625 ± 0.11	1.25 ± 0.20	5 ± 0.37	6.25 ± 0.18
**6cc**	1 ± 0.40	10 ± 0.14	1 ± 0.35	90	1.25 ± 0.46	1.25 ± 0.20	>5	12.5 ± 0.28
**6ce**	1 ± 0.40	1 ± 0.36	10 ± 0.55	90	>5	1.25 ± 0.20	>5	25 ± 0.26
**6cf**	1 ± 0.38	10 ± 0.14	1 ± 0.35	90	1.25 ± 0.14	1.25 ± 0.40	>5	12.5 ± 0.62
**6cg**	1 ± 0.20	10 ± 0.31	1 ± 0.44	90	1.25 ± 0.24	1.25 ± 0.25	>5	12.5 ± 0.29
**6ch**	1 ± 0.15	1 ± 0.25	10 ± 0.35	95	0.625 ± 0.07	1.25 ± 0.11	5 ± 0.27	6.25 ± 0.07
**6ci**	1 ± 0.39	1 ± 0.24	10 ± 0.39	90	>5	1.25 ± 0.31	>5	25 ± 0.42
Isoniazid	0.7 ± 0.05	50 ± 0.25	12.5 ± 0.34	95	0.7 ± 0.08	50 ± 0.21	12.5 ± 0.18	12.5 ± 0.20
Rifampicin	0.5 ± 0.35	1.5 ± 0.38	1.5 ± 0.34	95	0.5 ± 0.41	1.5 ± 0.16	1.5 ± 0.22	25 ± 0.21

^a^ *Mycobacterium tuberculosis* H37Rv; ^b^ *Mycobacterium smegmatis* (ATCC 19420); ^c^ *Mycobacterium fortuitum* (ATCC 19542); ^d^ percentage of inhibition against *M. tuberculosis* H37Rv; ‘-’ not detected. The MIC values were determined in triplicate and expressed as the mean ± SD.

**Table 4:** Calculated LogP and CLogP values for the synthesized compounds **6aa–6ci**.

Compounds	LogP	CLogP
**6aa**	2.30	1.7159
**6ab**	2.58	1.9399
**6ac**	2.74	2.1059
**6ad**	0.69	0.9292
**6af**	0.91	0.6929
**6ag**	2.93	3.1377
**6ai**	2.24	2.1239
**6bb**	2.45	1.9840
**6bc**	2.61	2.1500
**6be**	2.30	1.8410
**6bf**	0.78	0.7370
**6bg**	2.80	3.0567
**6bh**	2.78	2.7994
**6bi**	2.11	2.1680
**6cb**	3.57	2.9459
**6cc**	3.73	3.1119
**6ce**	3.41	2.9280
**6cf**	1.89	1.8240
**6cg**	3.91	4.1437
**6ch**	3.90	3.8864
**6ci**	3.22	3.2550

## Data Availability

Data are contained within the article and Supplementary Materials.

## Supplementary Materials

The following supporting information can be downloaded at: https://www.mdpi.com/article/10.3390/ph17050548/s1, the NMR spectra of all the new compounds tested in this study.
