# Supplementary material for: NMI-SO2Cl2-Mediated Amide Bond Formation: Facile Synthesis of Some Dihydrotriazolopyrimidine Amide Derivatives as Potential Anti-Inflammatory and Anti-Tubercular Agents
Source: Pharmaceuticals (Basel). 2024 Apr 24;17(5):548. doi: 10.3390/ph17050548 (PMC11123900; doi:10.3390/ph17050548)

Supporting Information

NMI-SO_2Cl_2-Mediated Amide Bond Formation: Facile Synthesis of Some Dihydrotriazolopyrimidine Amide Derivatives as Potential Anti-Inflammatory and Anti-Tubercular Agents

Aravinda Babu [1], Kenchaiah Sunil [1], Ayyiliath Meleveetil Sajith [1], Eeda Koti Reddy [2], Sougata Santra [3], Grigory V. Zyryanov [3,4], Talavara Venkatesh [5], Bhadrachari Somashekara [6] and Nibin Joy Muthipeedika [3,*]

[1] Department of Chemistry, SSIT, Sri Siddhartha Academy of Higher Education, Tumkur 572107, Karnataka, India

[2] Department of Chemistry, Vignan's Foundation for Science, Technology and Research–VFSTR (Deemed to be University), Vadlamudi, Guntur 522213, Andhra Pradesh, India

[3] Laboratory of Organic Synthesis, Institute of Chemical Technology, Ural Federal University, 19 Mira Street, Yekaterinburg 620002, Russia

[4] I. Ya. Postovskiy Institute of Organic Synthesis, Ural Division of the Russian Academy of Sciences, 22 S. Kovalevskoy Street, Yekaterinburg 620219, Russia

[5] Department of P.G studies and Research in Chemistry, Kuvempu University, Jnana Sahyadri, Shankaraghatta, Shimoga 577451, Karnataka, India

[6] Department of Chemistry, Smt. Indira Gandhi Government First Grade Women's College, Sagar 577401, Karnataka, India

* Correspondence: mnibinjoy@gmail.com

^{1}H NMR of Compound of **4a**

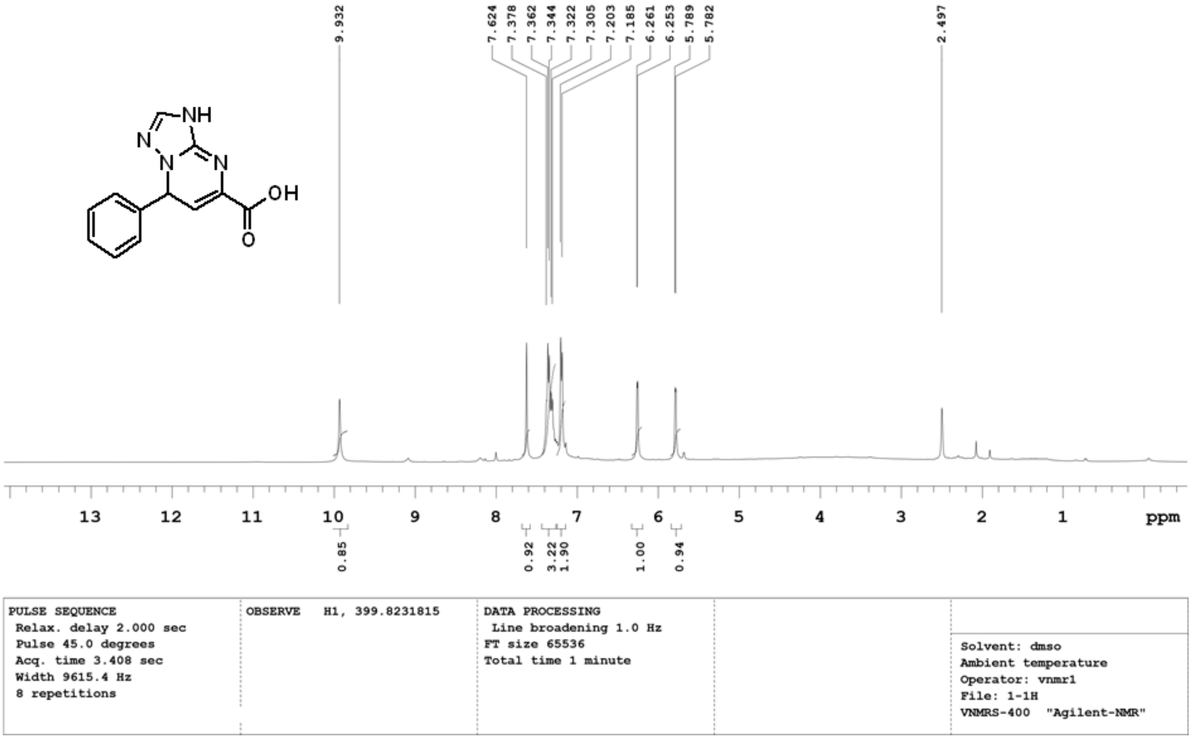

^{13}C NMR of Compound of **4a**

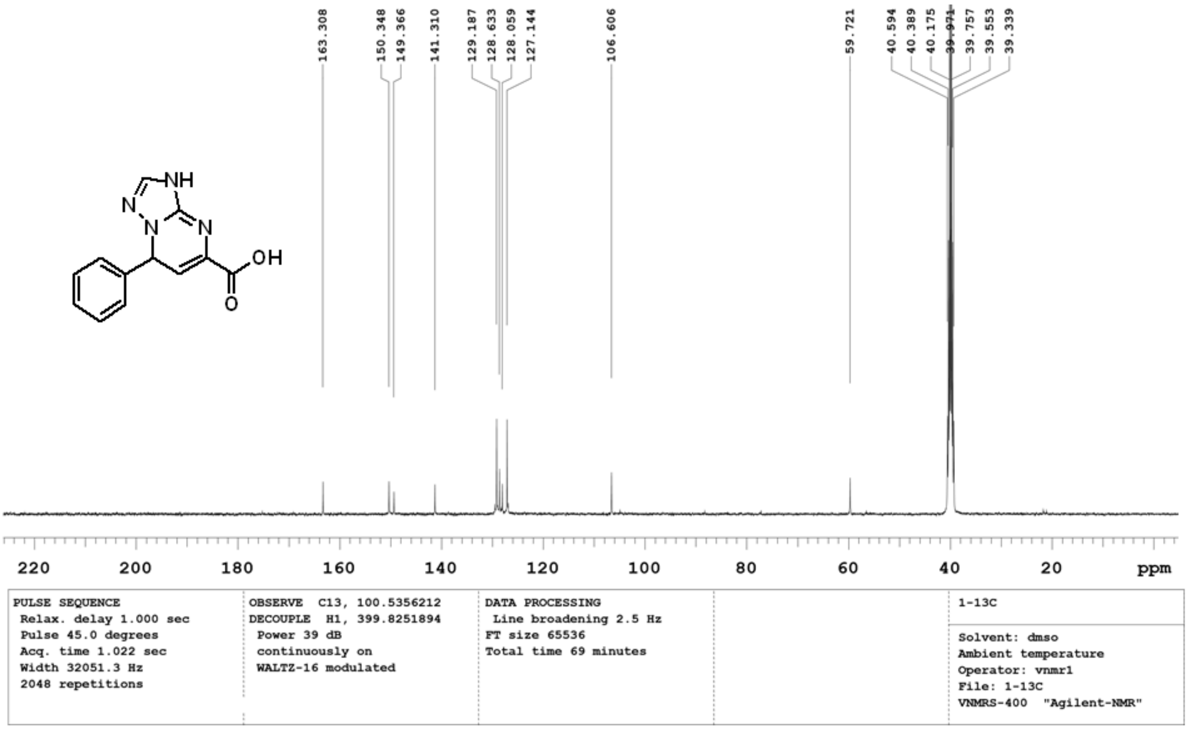

^{1}H NMR of Compound of **6aa**

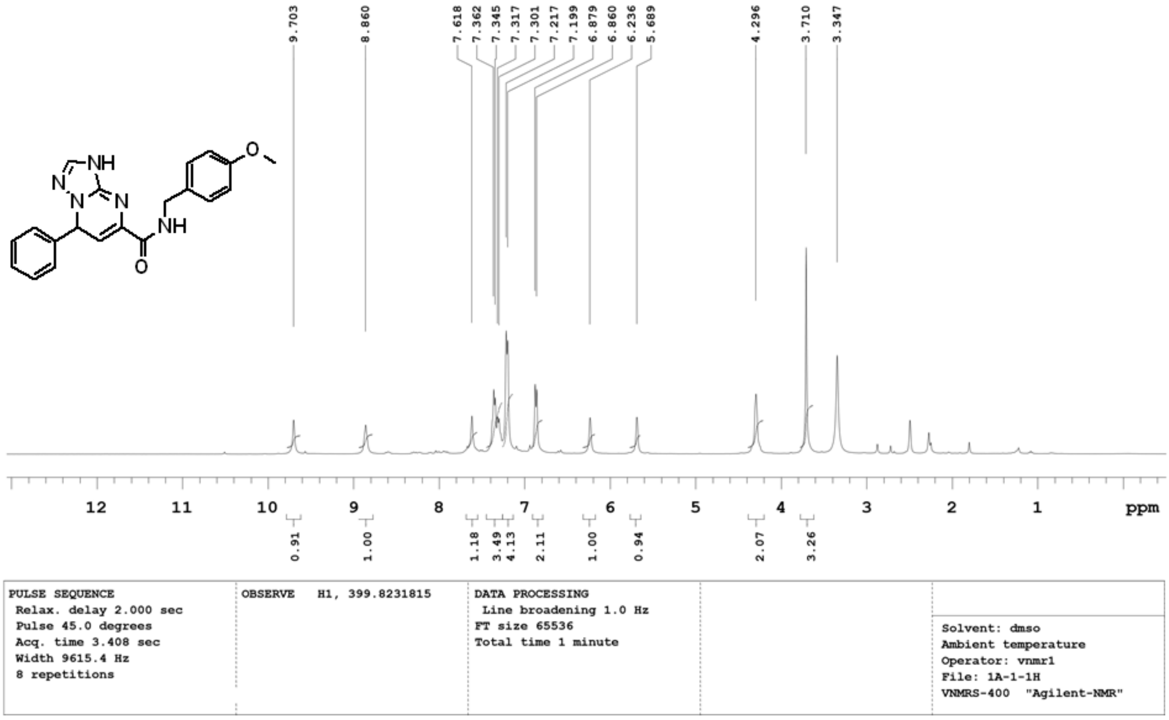

^{13}C NMR of Compound of **6aa**

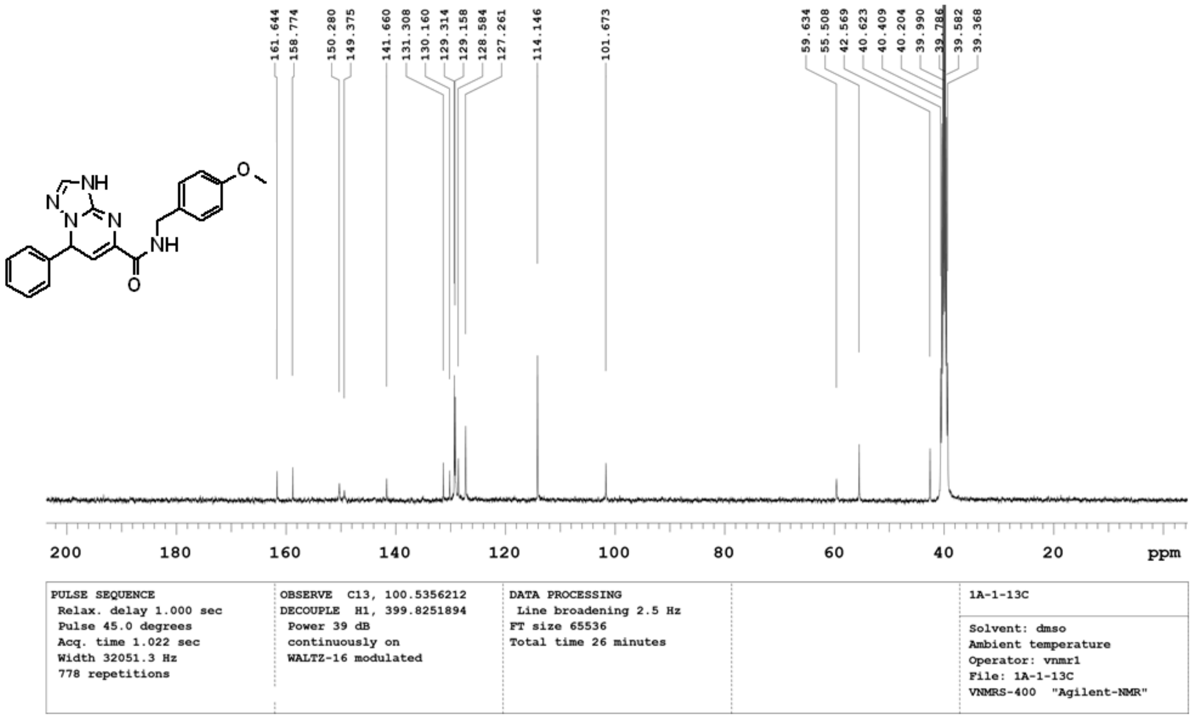

^{1}H NMR of Compound of **6ab**

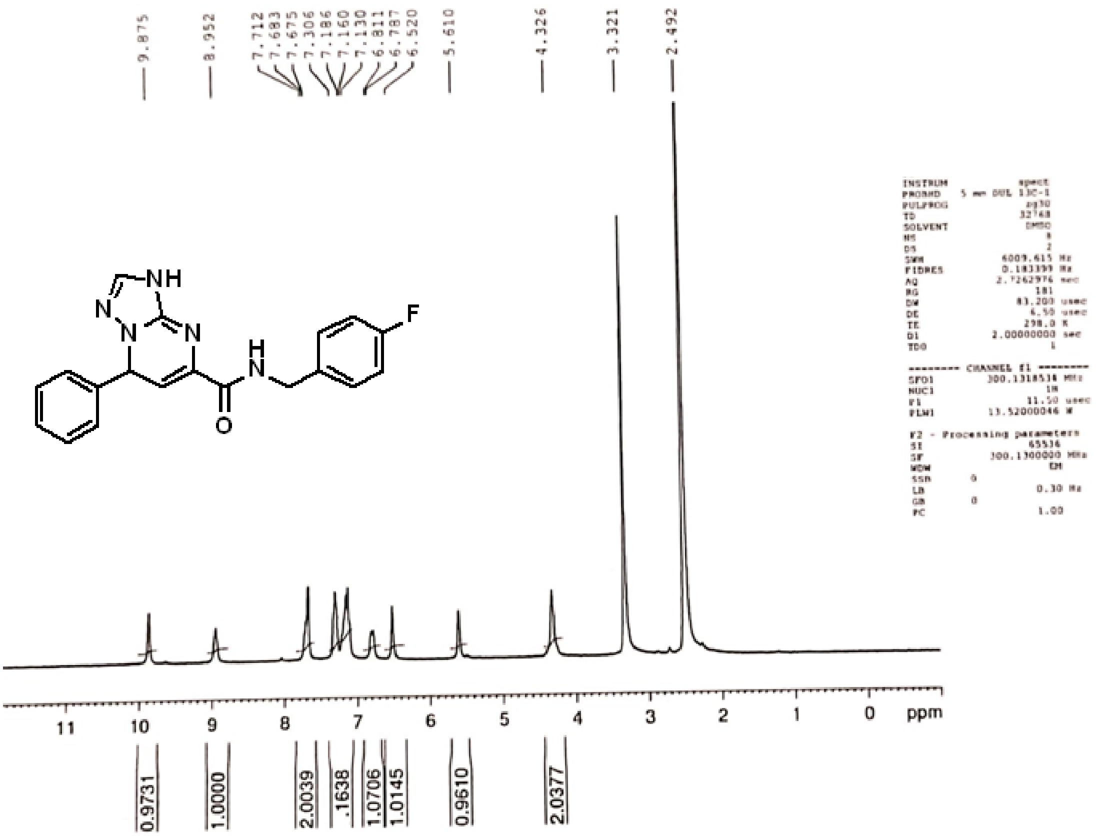

^{13}C NMR of Compound of **6ab**

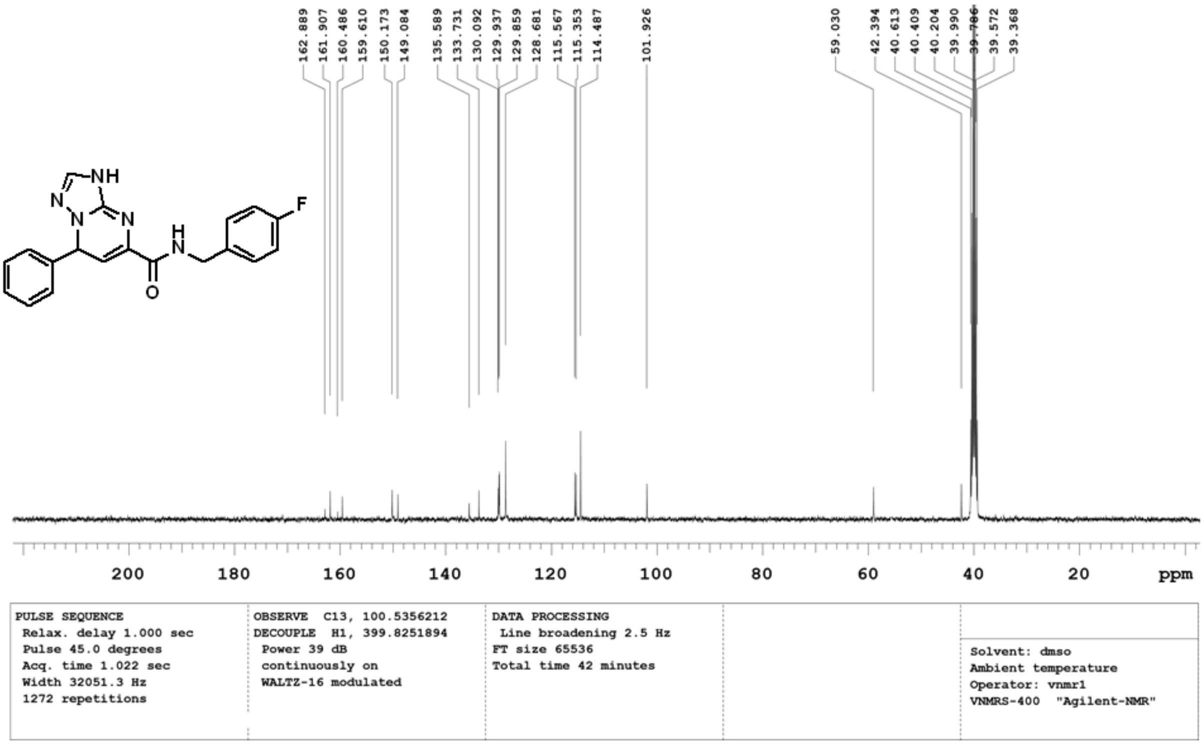

^{1}H NMR of Compound of **6ac**

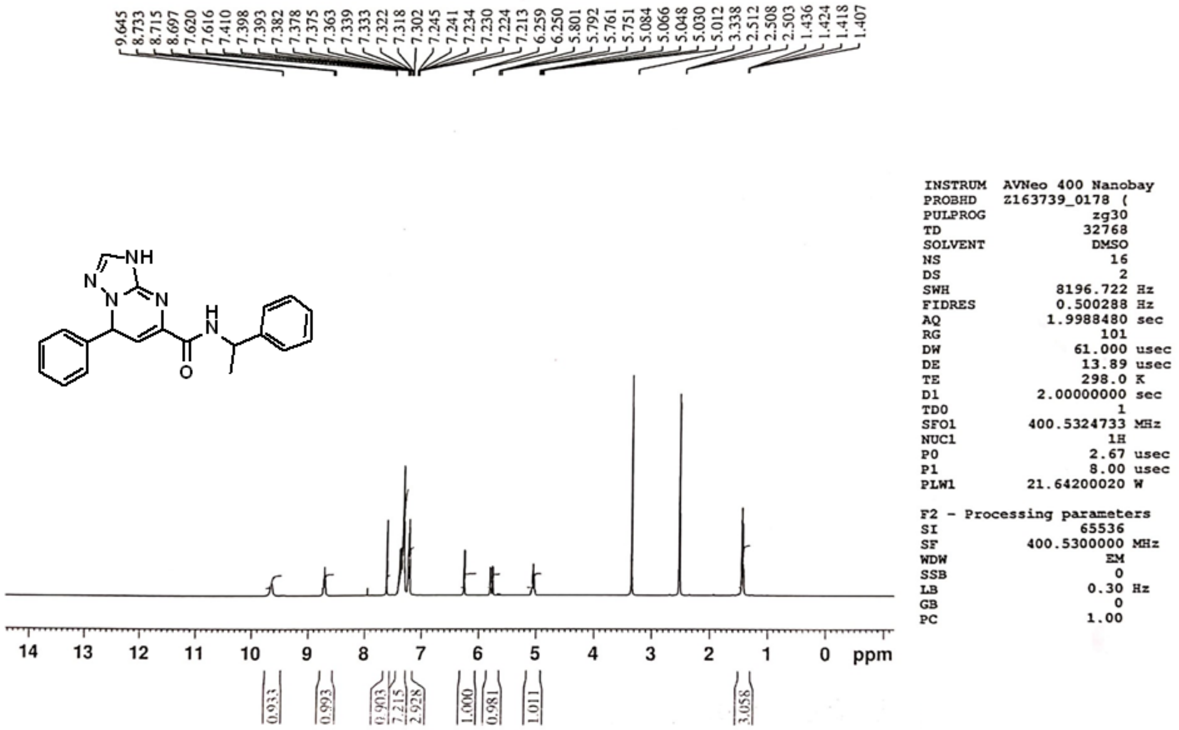

^{13}C NMR of Compound of **6ac**

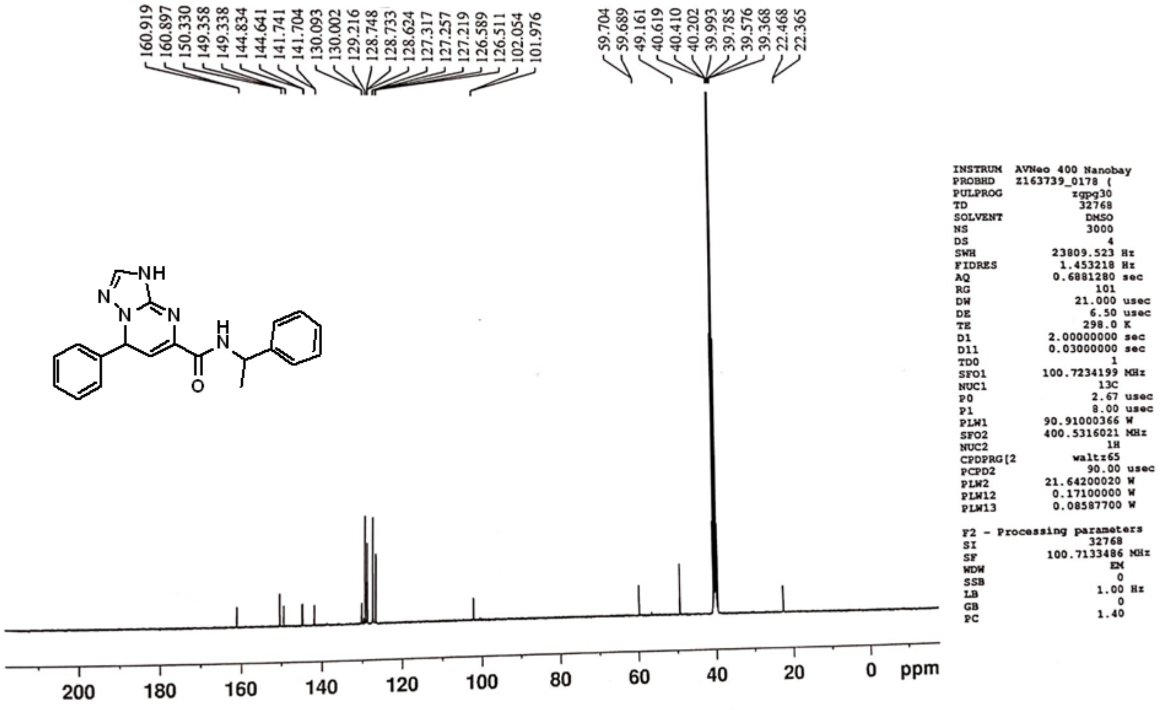

^{1}H NMR of Compound of **6ad**

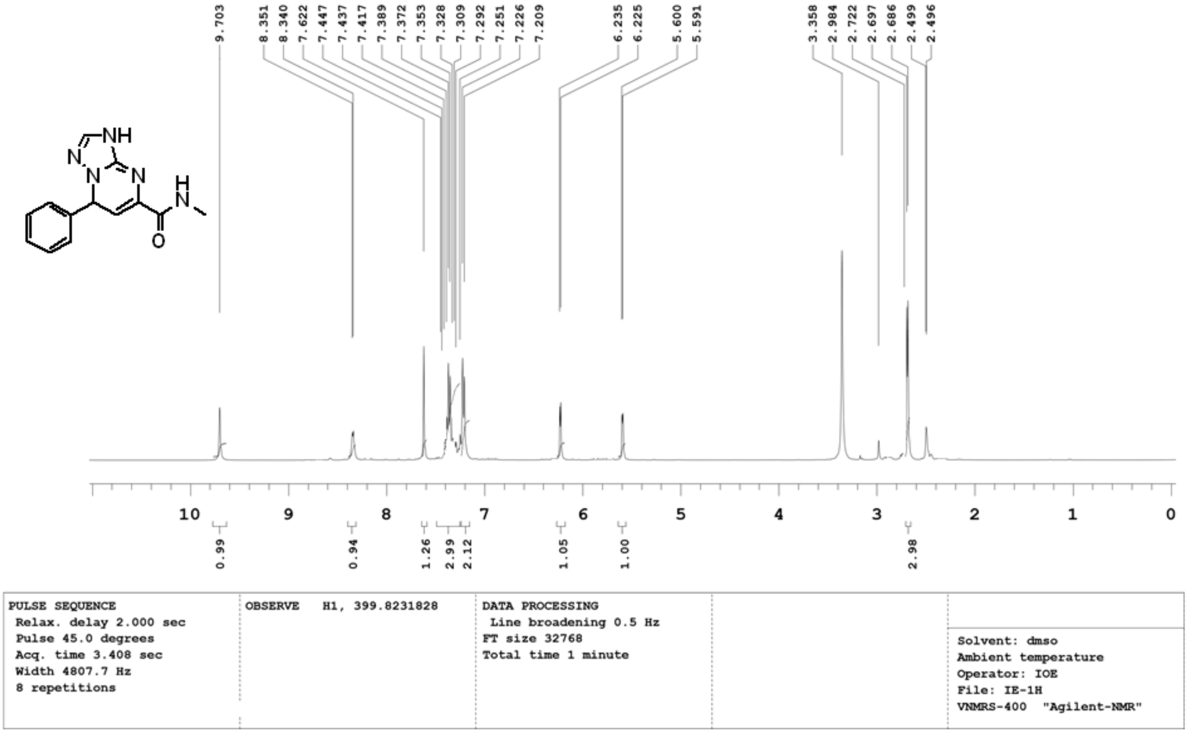

^{13}C NMR of Compound of **6ad**

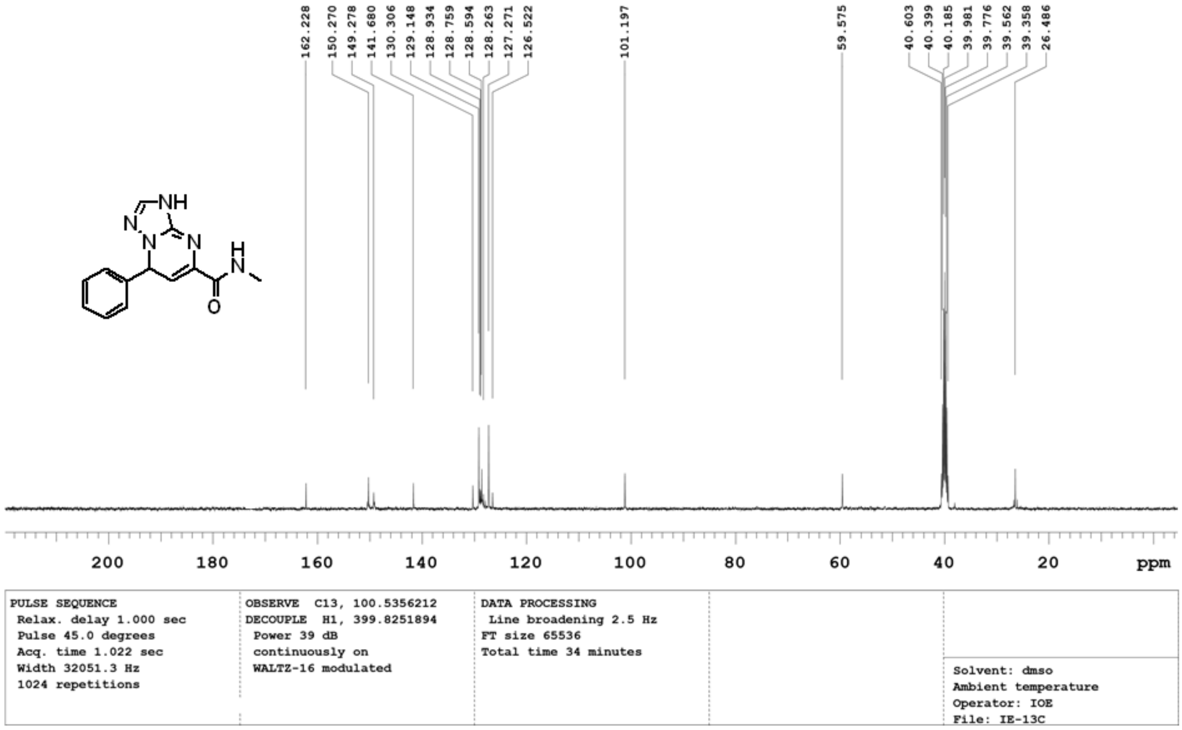

^{1}H NMR of Compound of **6af**

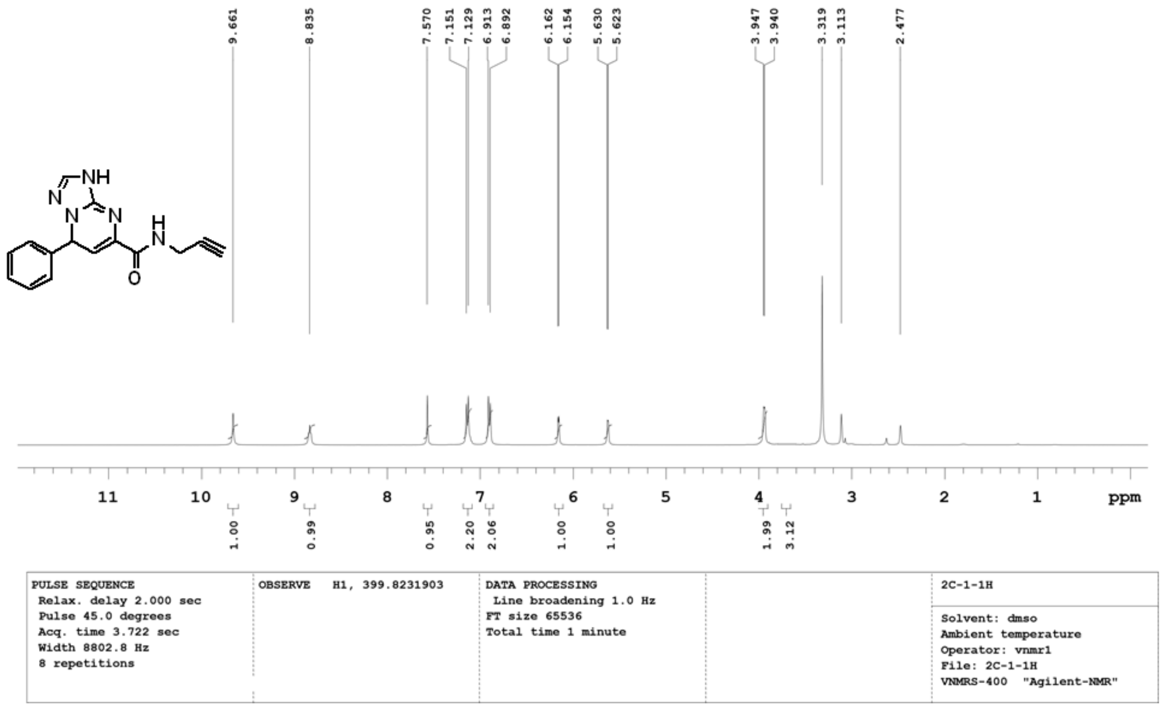

^{13}C NMR of Compound of **6af**

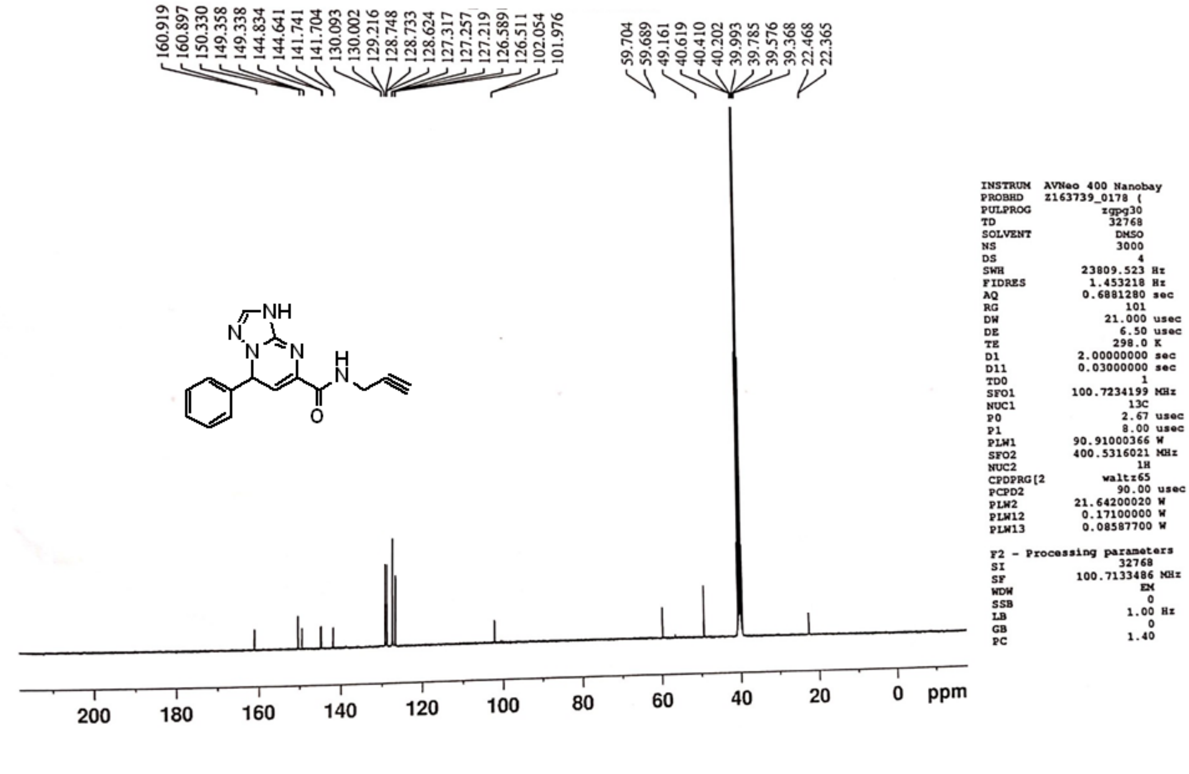

^{1}H NMR of Compound of **6ag**

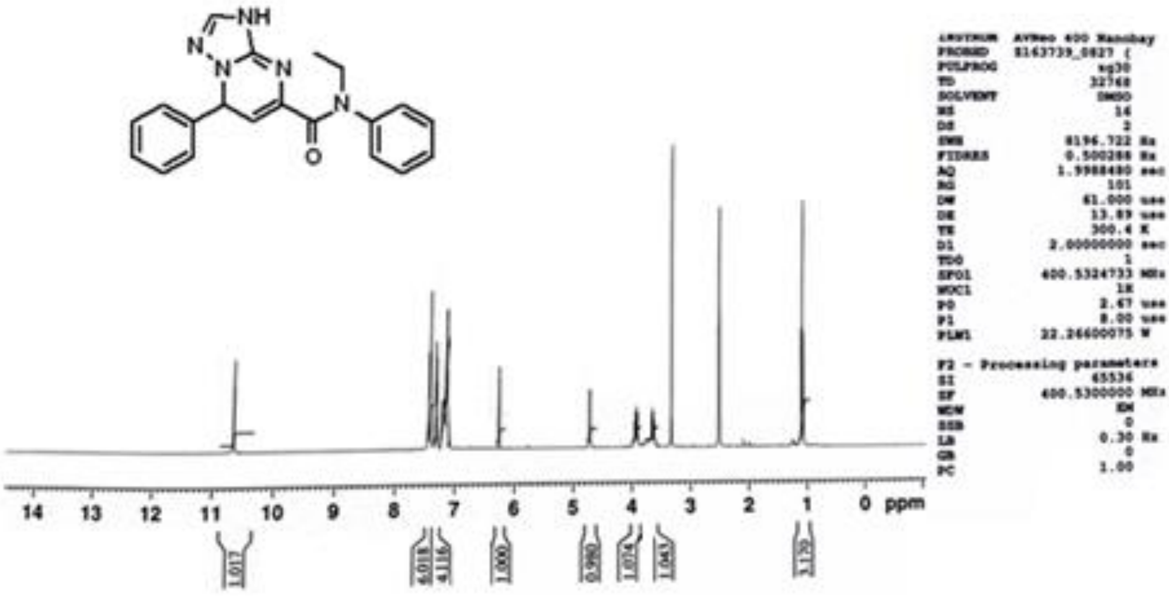

^{13}C NMR of Compound of **6ag**

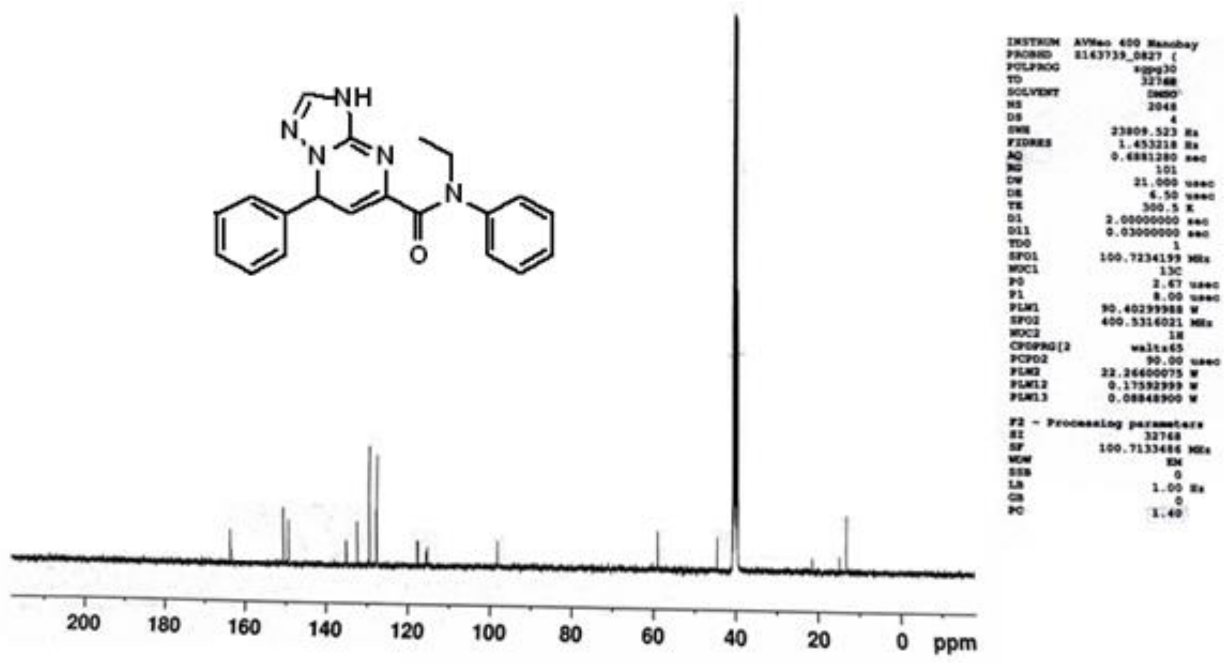

^{1}H NMR of Compound of **6ai**

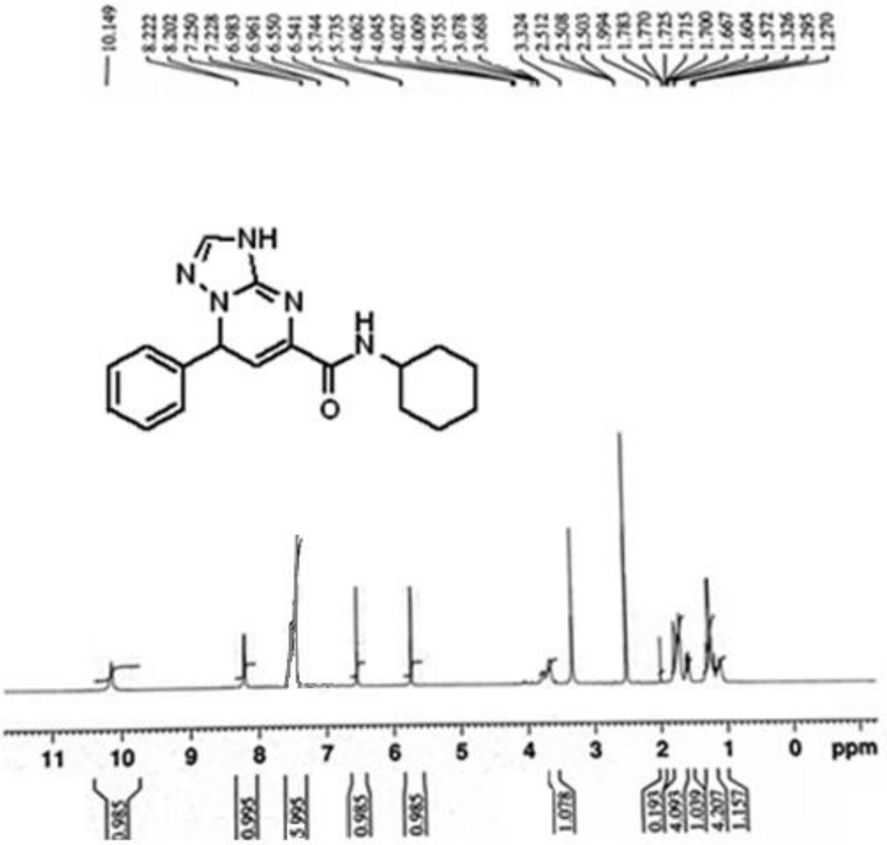

^{13}C NMR of Compound of **6ai**

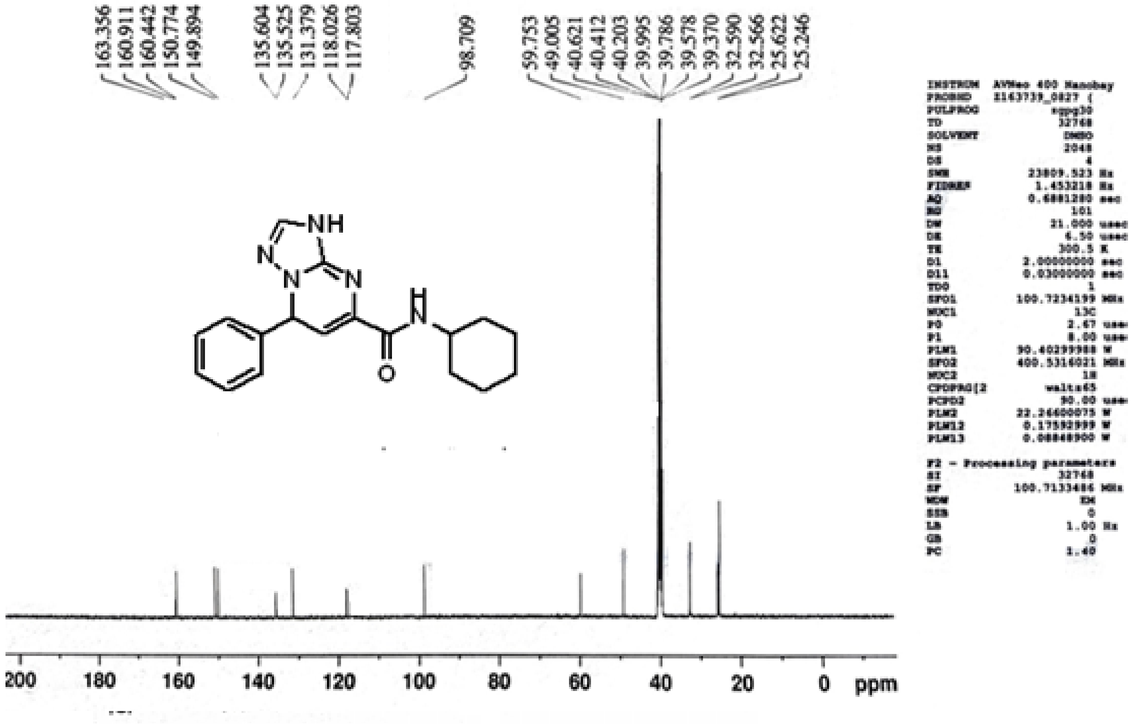

^{1}H NMR of Compound of **4b**

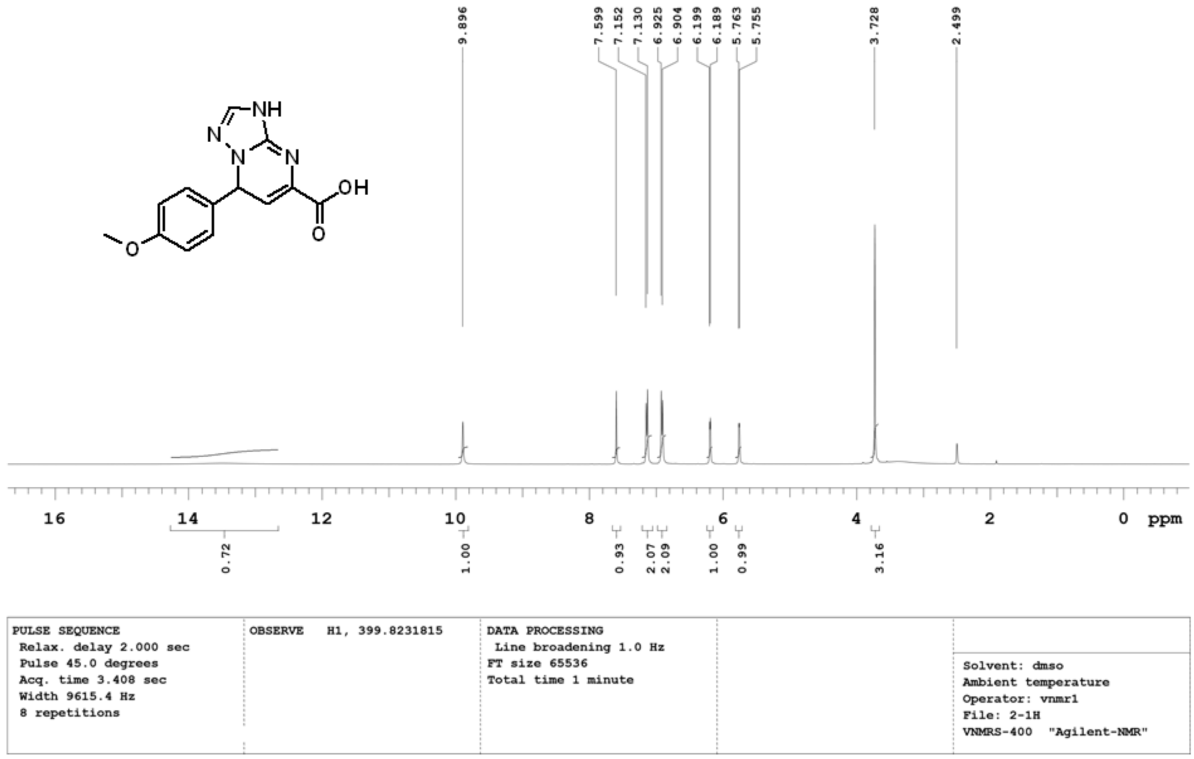

^{13}C NMR of Compound of **4b**

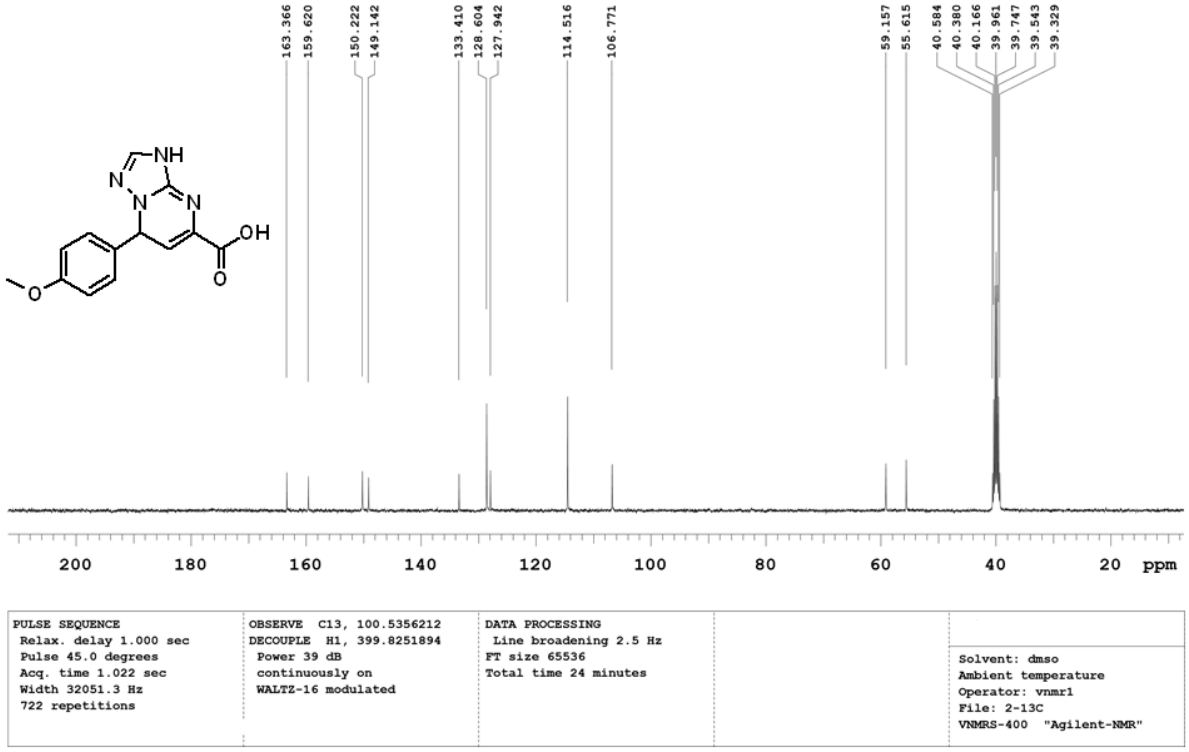

^{1}H NMR of Compound of **6bb**

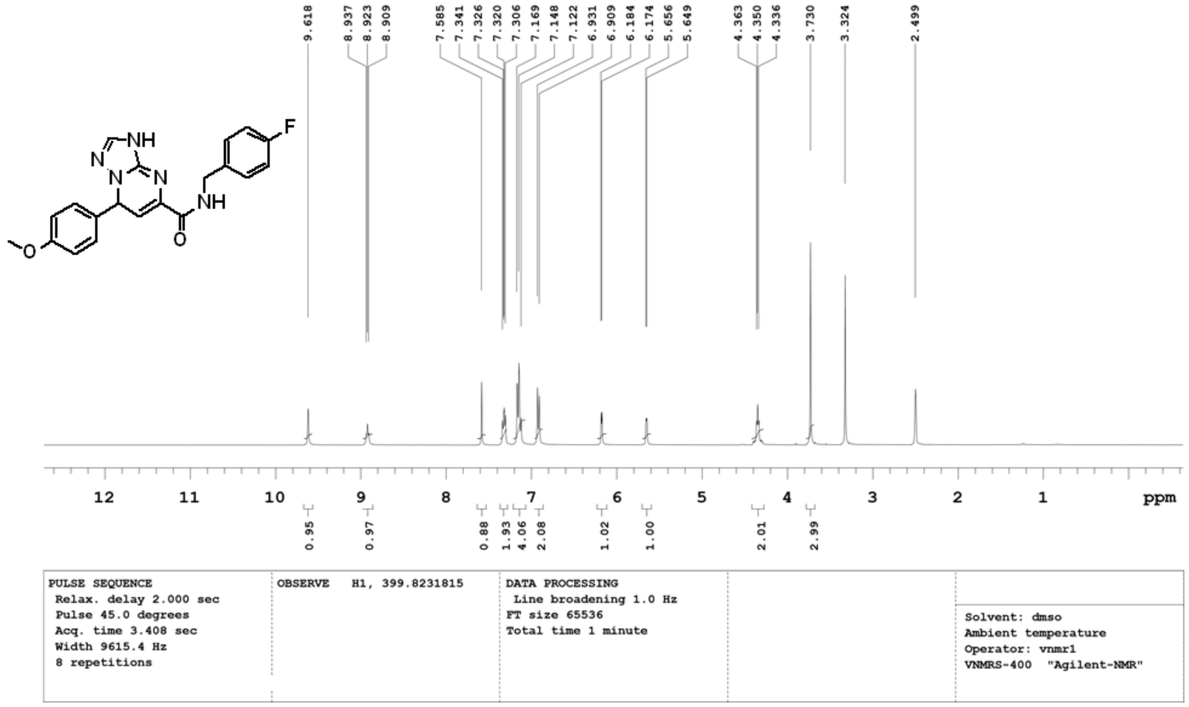

^{13}C NMR of Compound of **6bb**

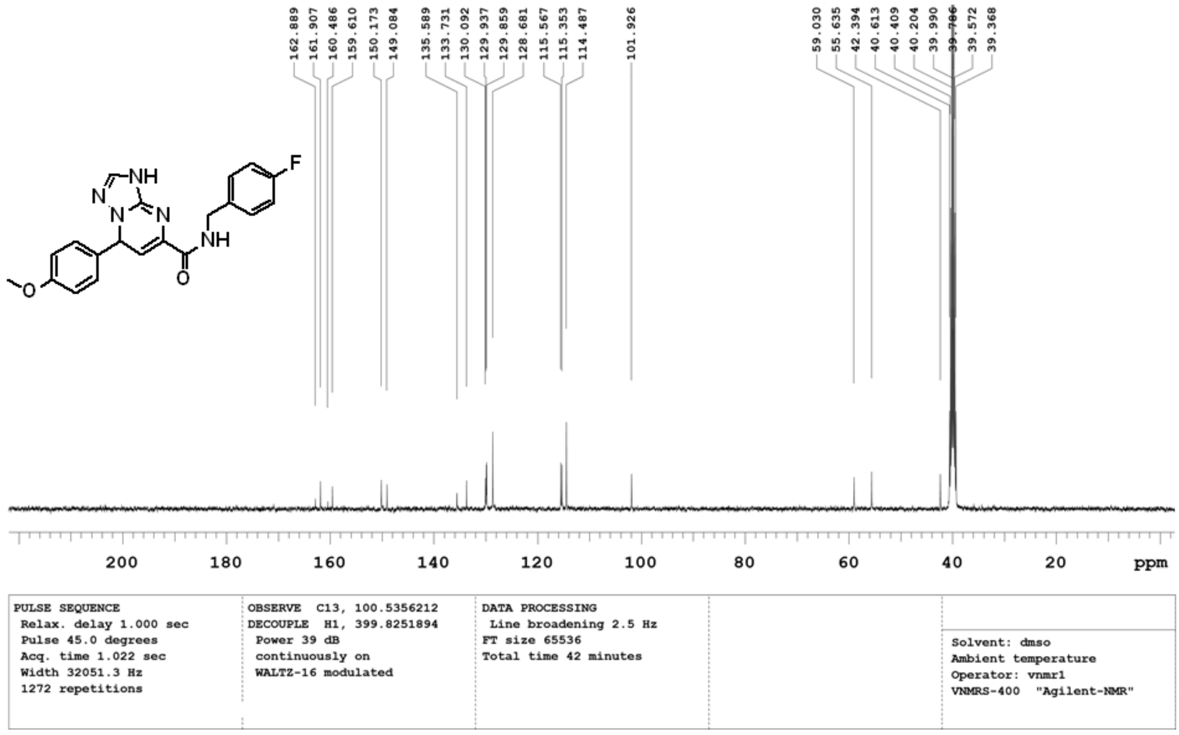

^{1}H NMR of Compound of **6bc**

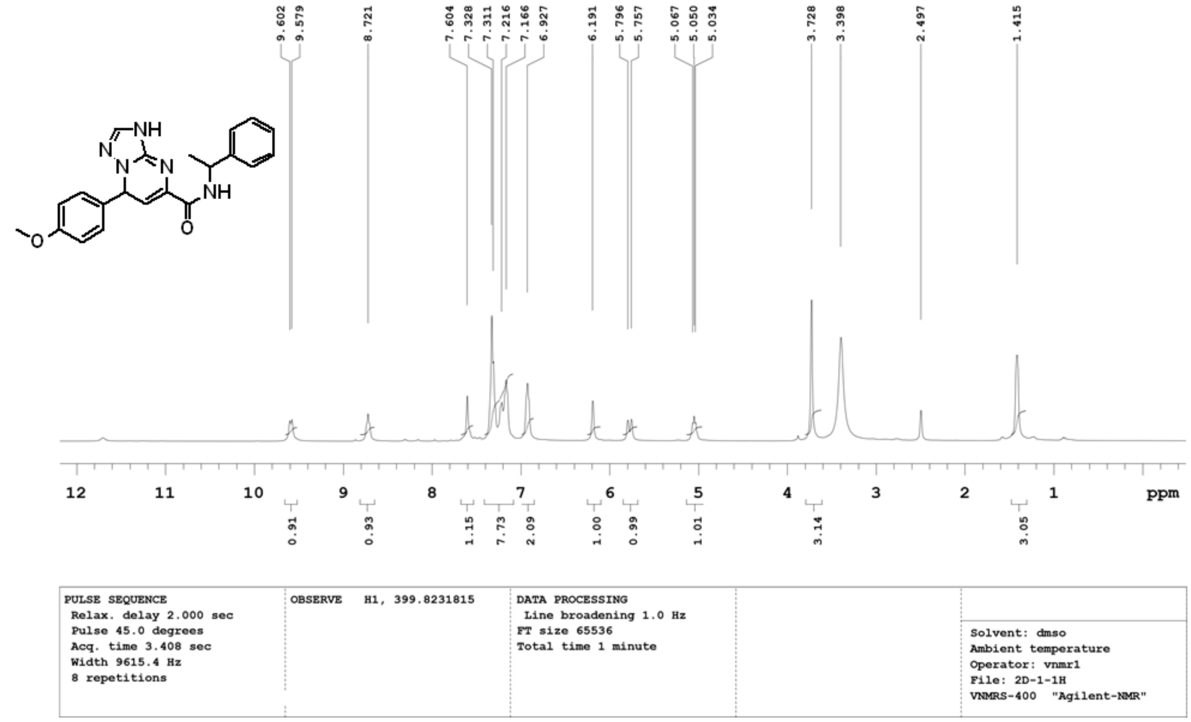

^{13}C NMR of Compound of **6bc**

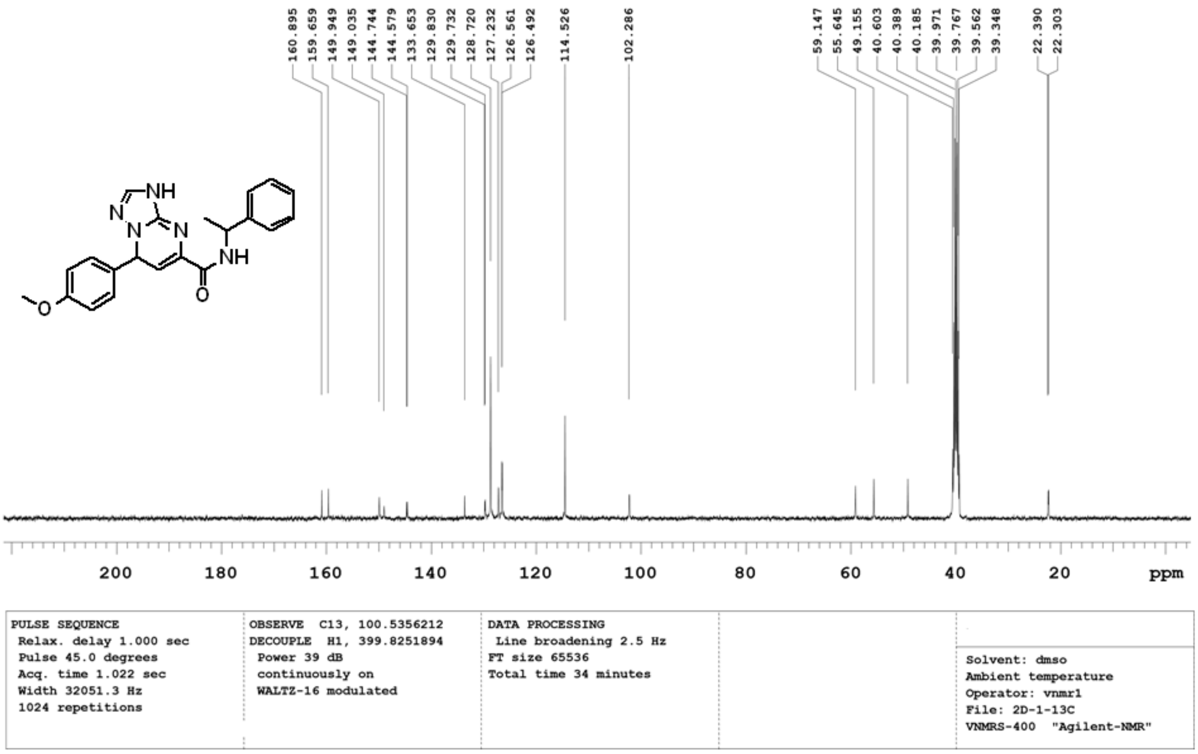

^{1}H NMR of Compound of **6be**

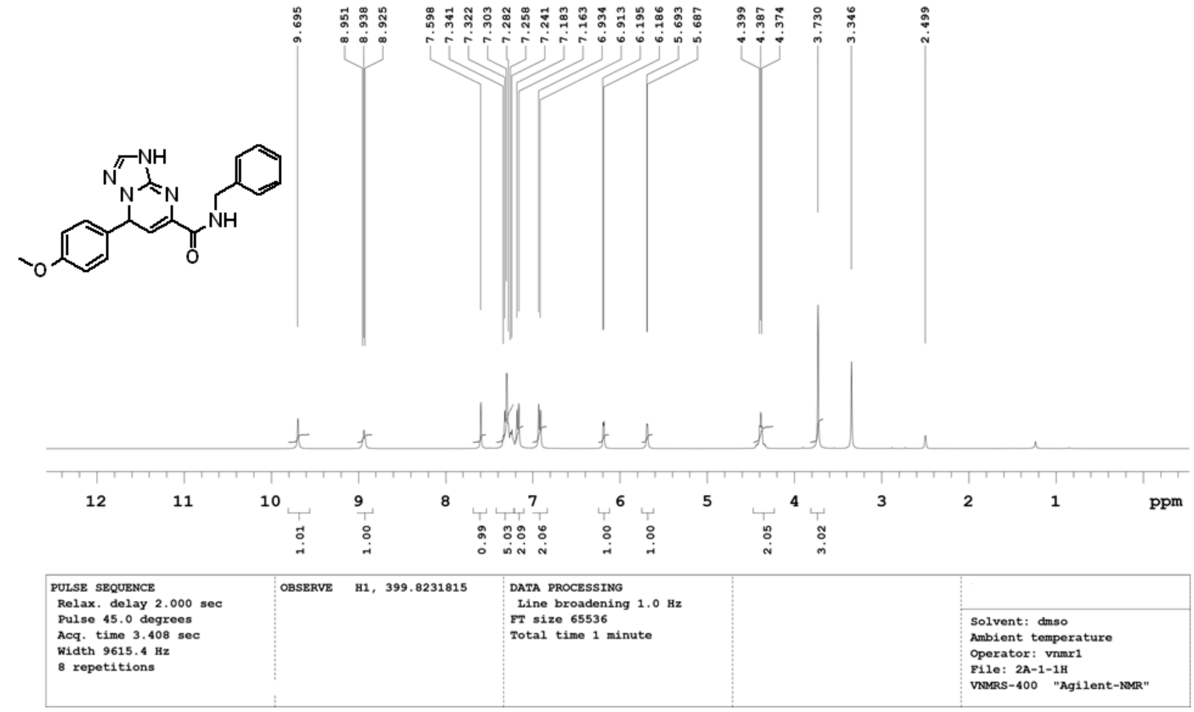

^{13}C NMR of Compound of **6be**

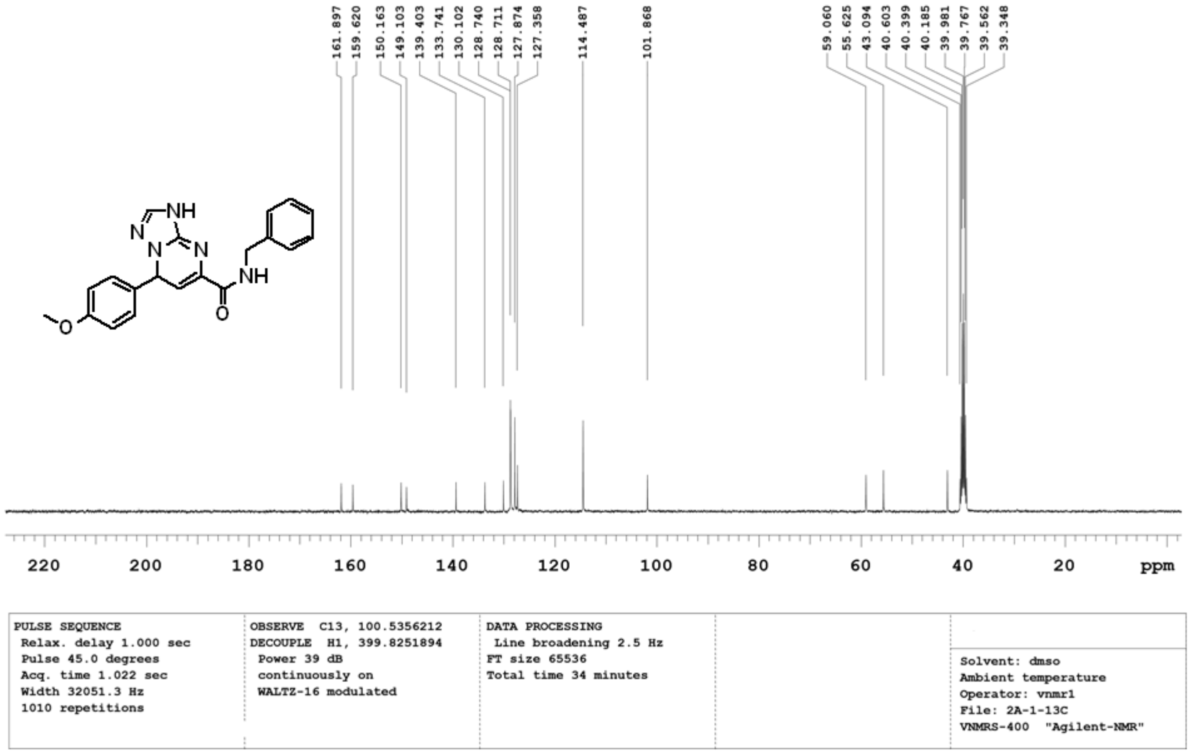

^{1}H NMR of Compound of **6bf**

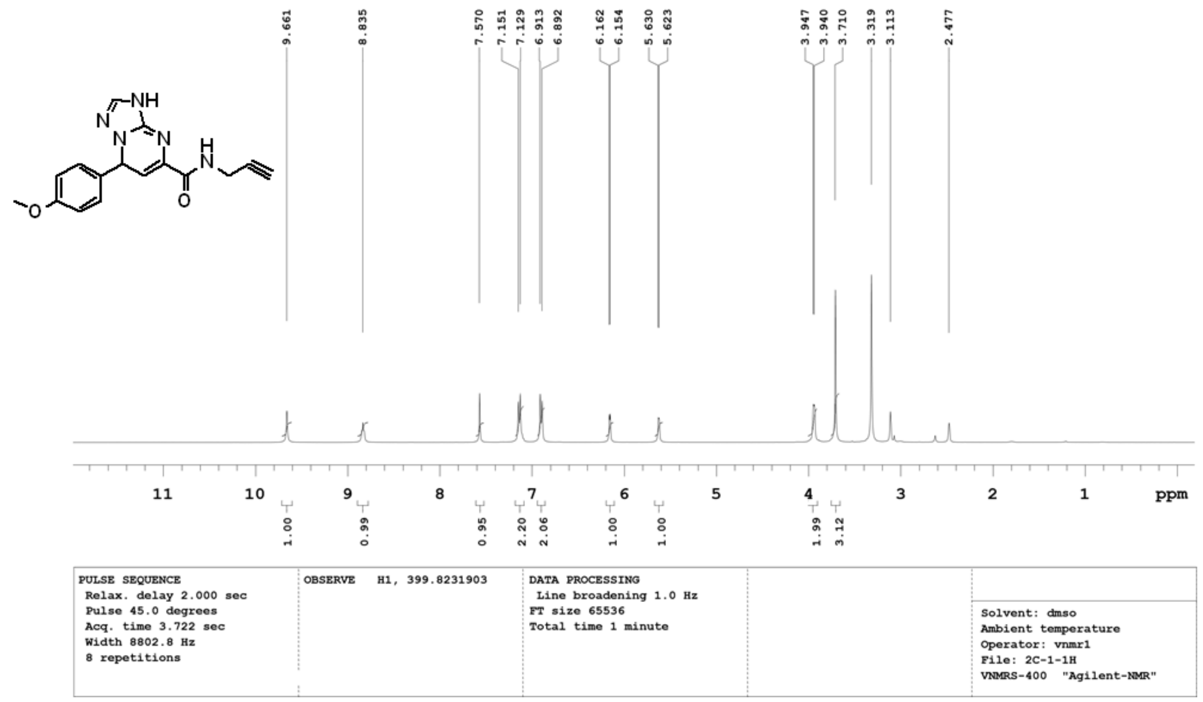

^{1}H NMR of Compound of **6bg**

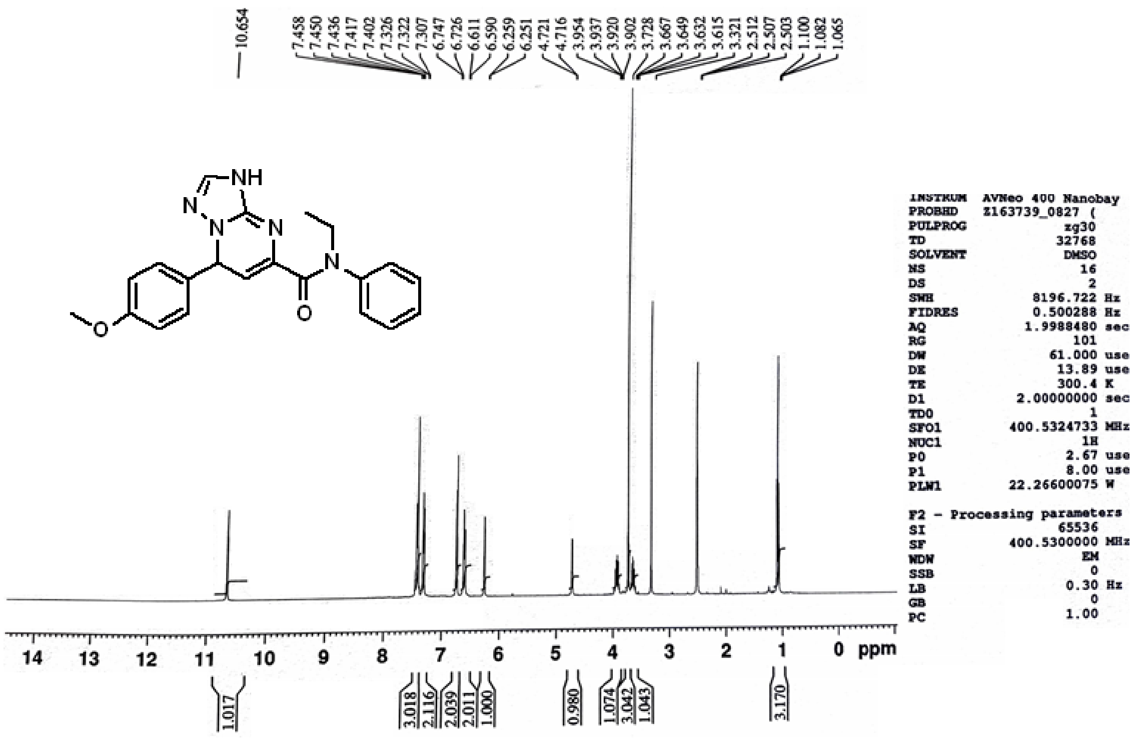

^{13}C NMR of Compound of **6bg**

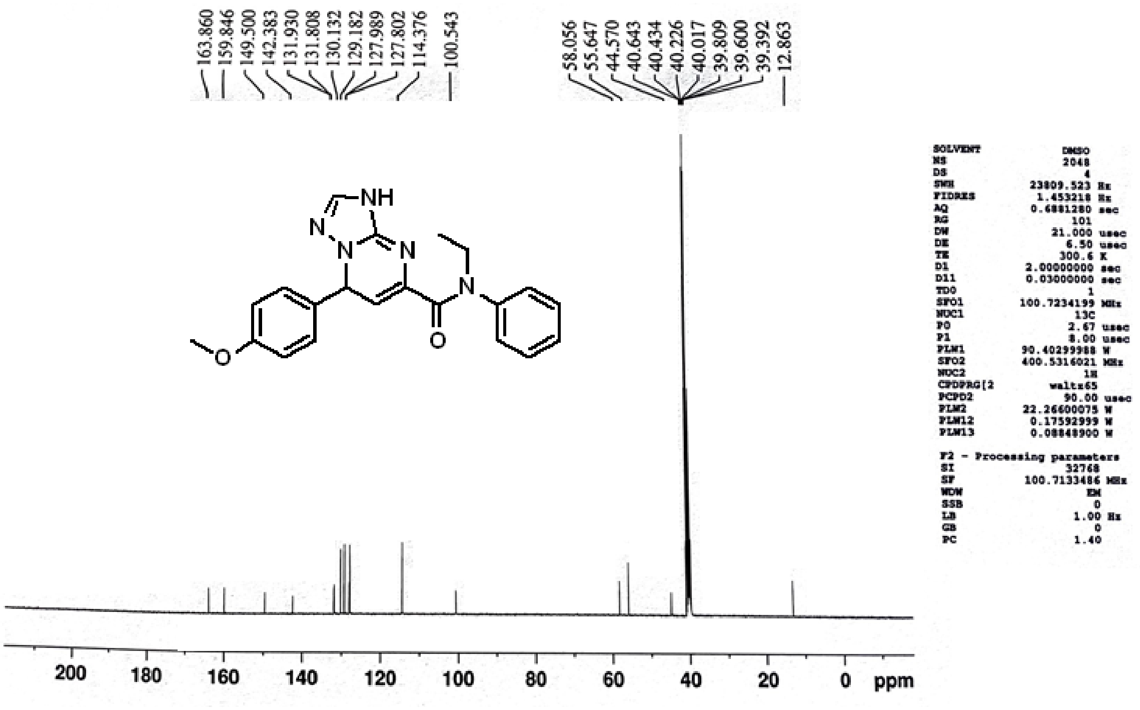

^{1}H NMR of Compound of **6bh**

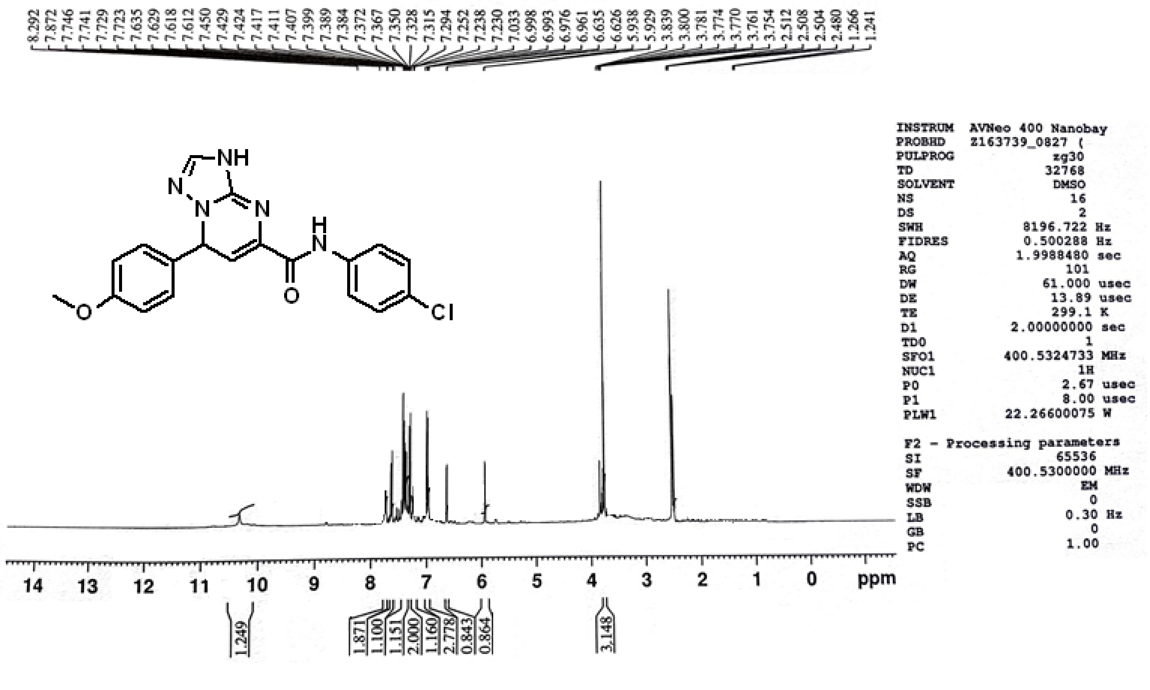

^{13}C NMR of Compound of **6bh**

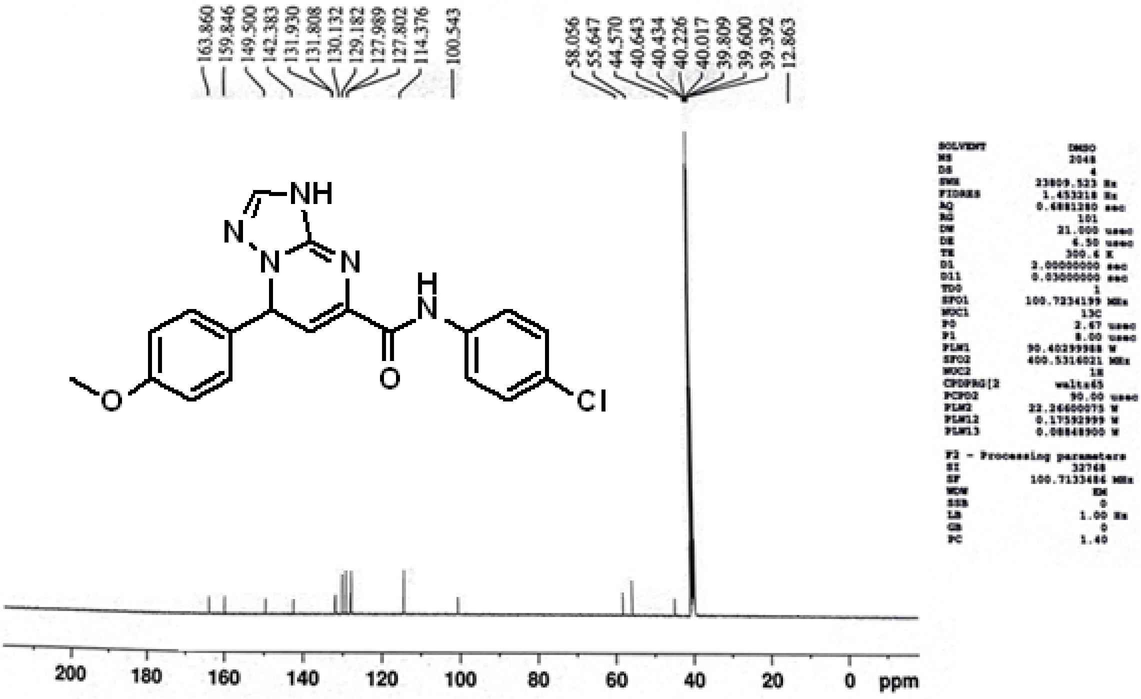

^{1}H NMR of Compound of **6bi**

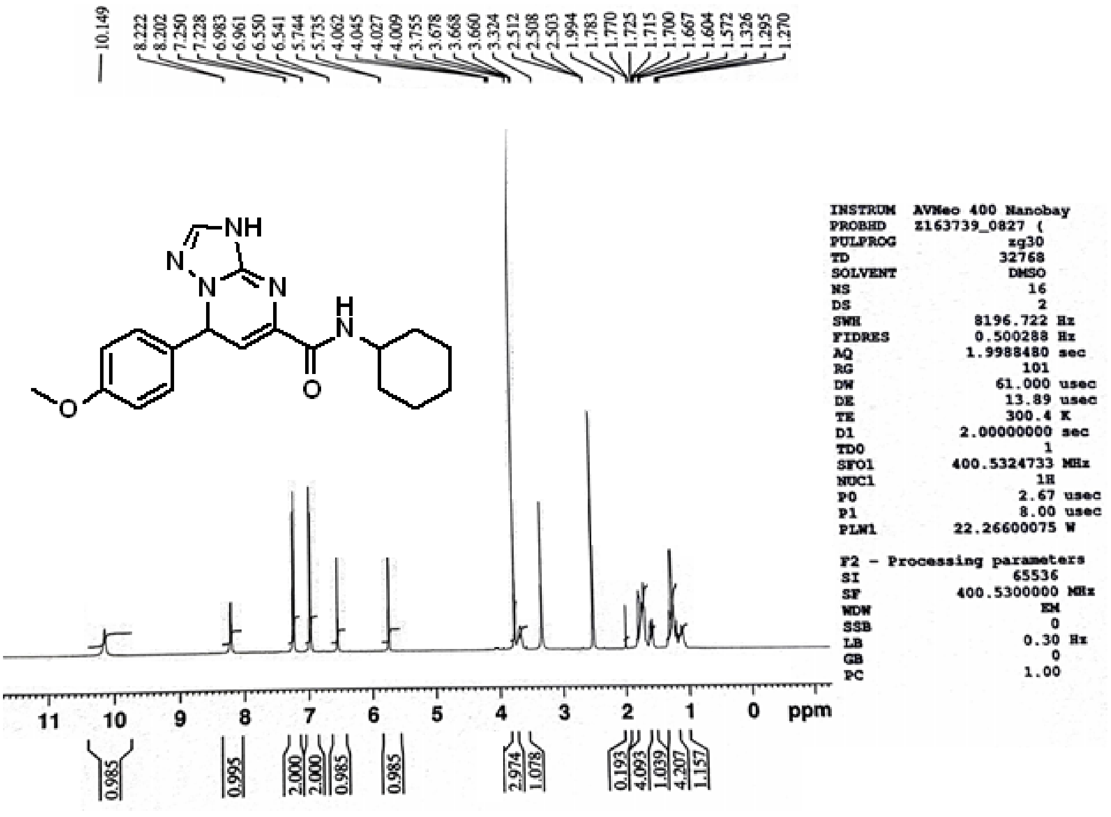

^{13}C NMR of Compound of **6bi**

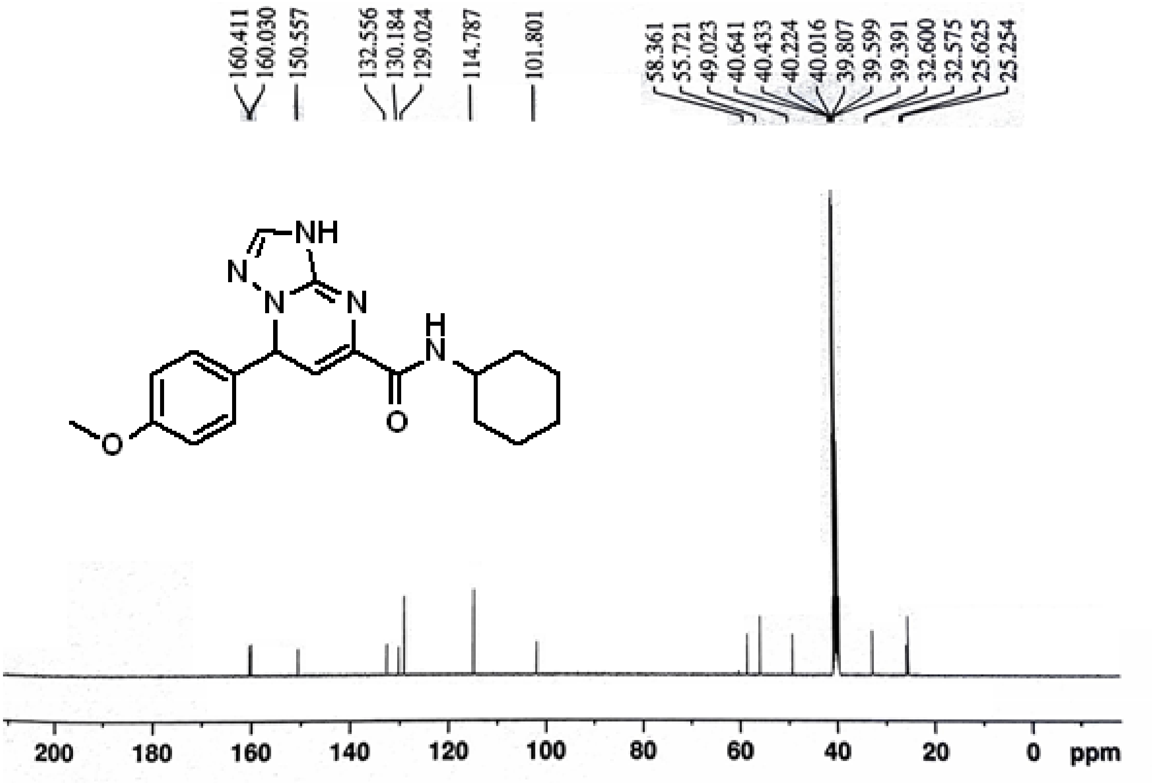

^{1}H NMR of Compound of **4c**

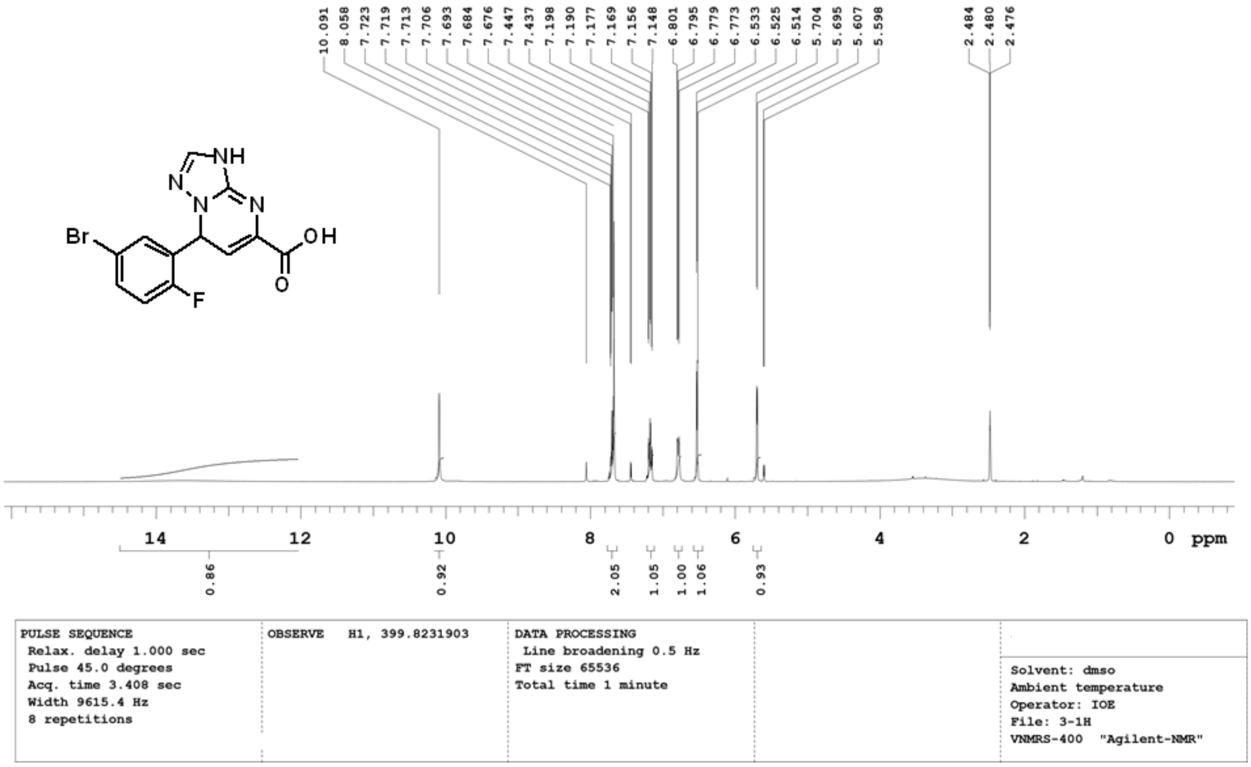

^{13}C NMR of Compound of **4c**

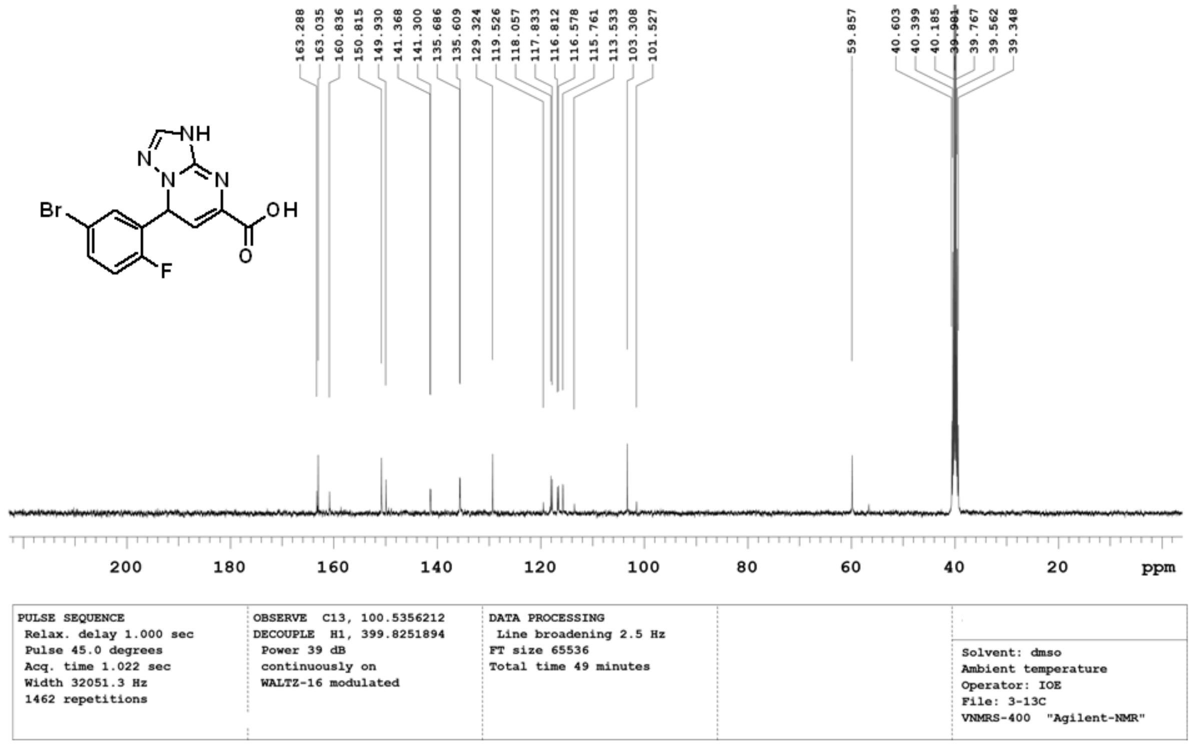

^{1}H NMR of Compound of **6cb**

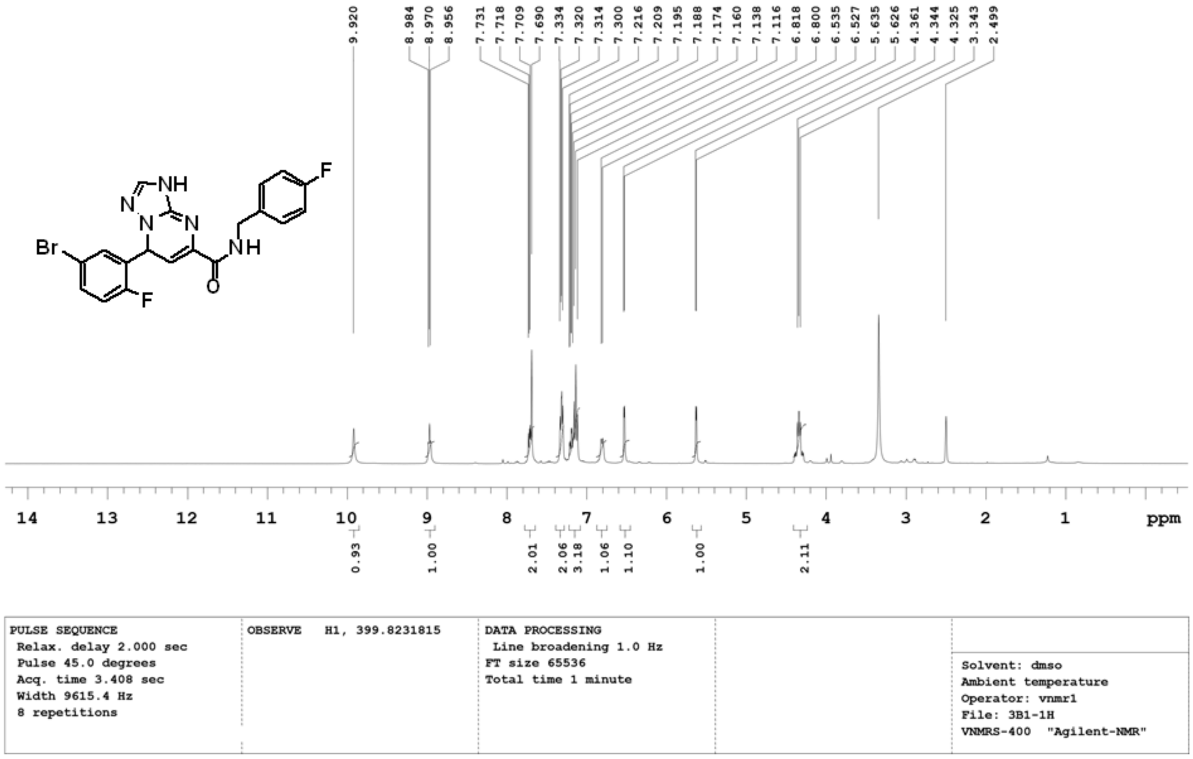

^{13}C NMR of Compound of **6cb**

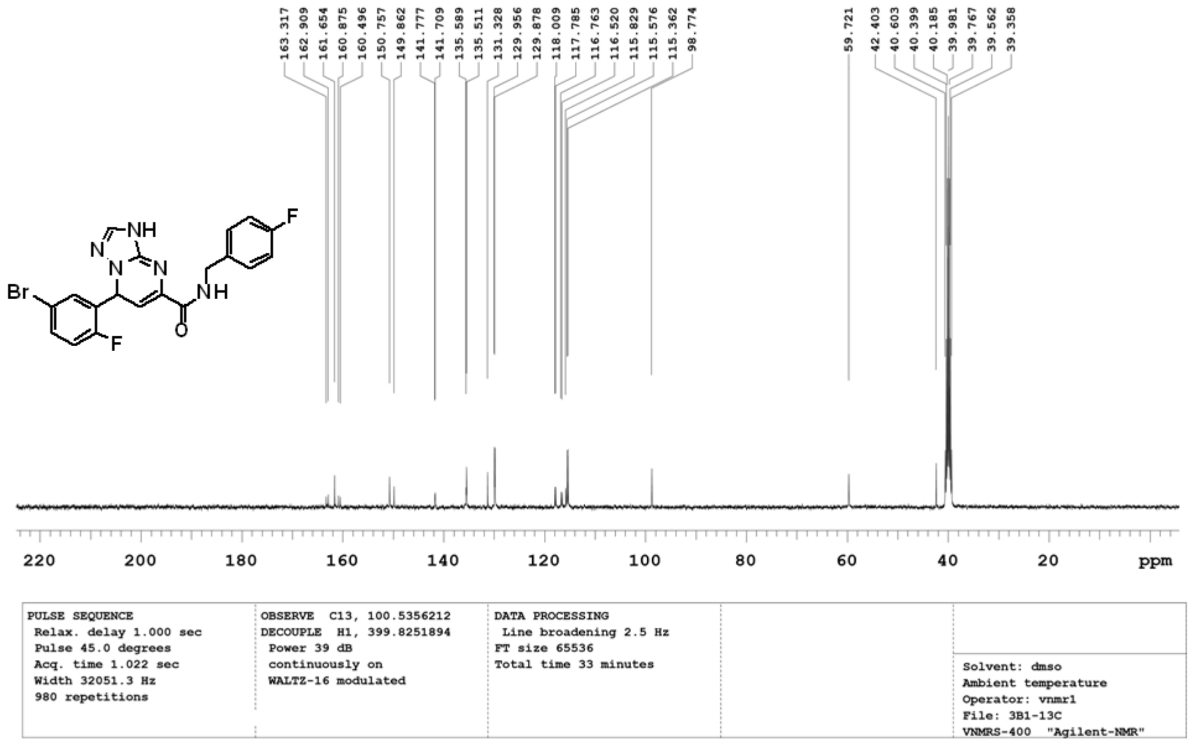

^{1}H NMR of Compound of **6cc**

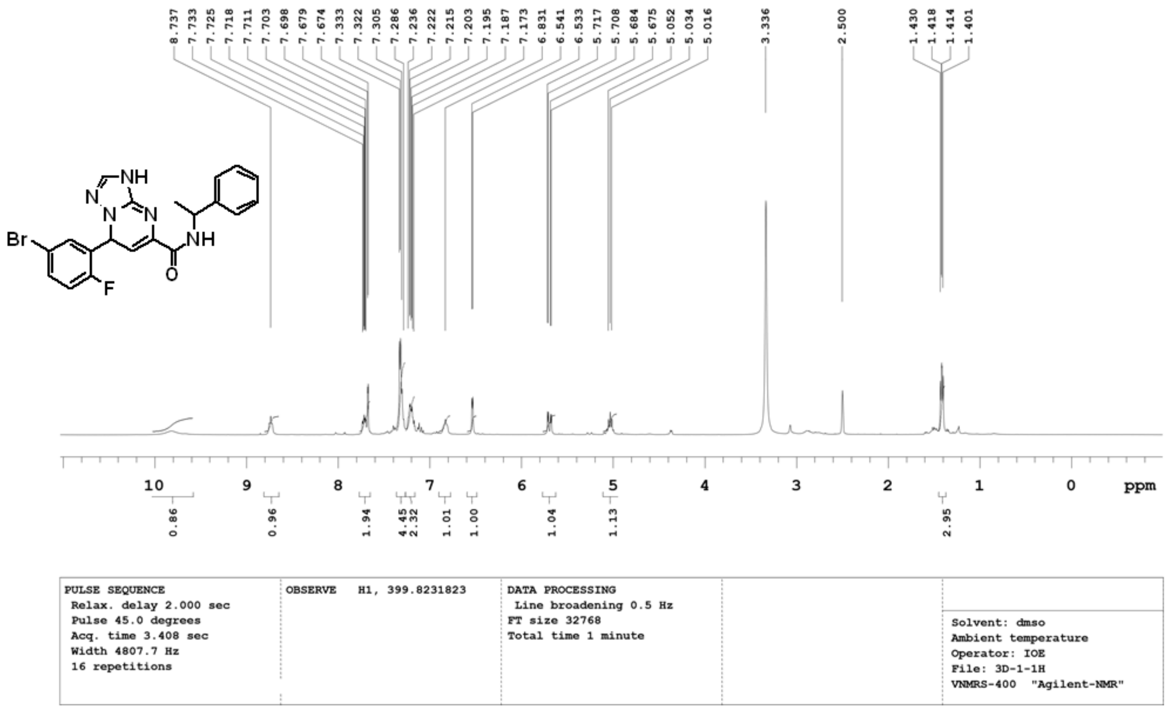

^{13}C NMR of Compound of **6cc**

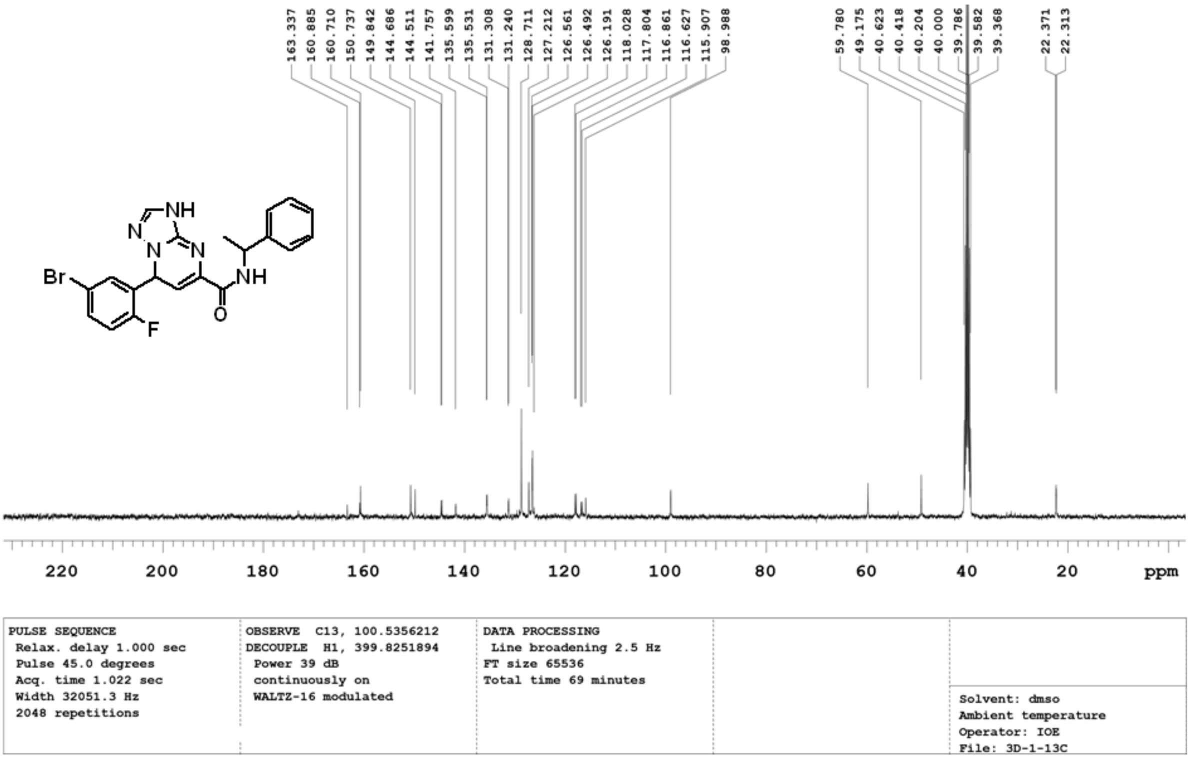

^{1}H NMR of Compound of **6ce**

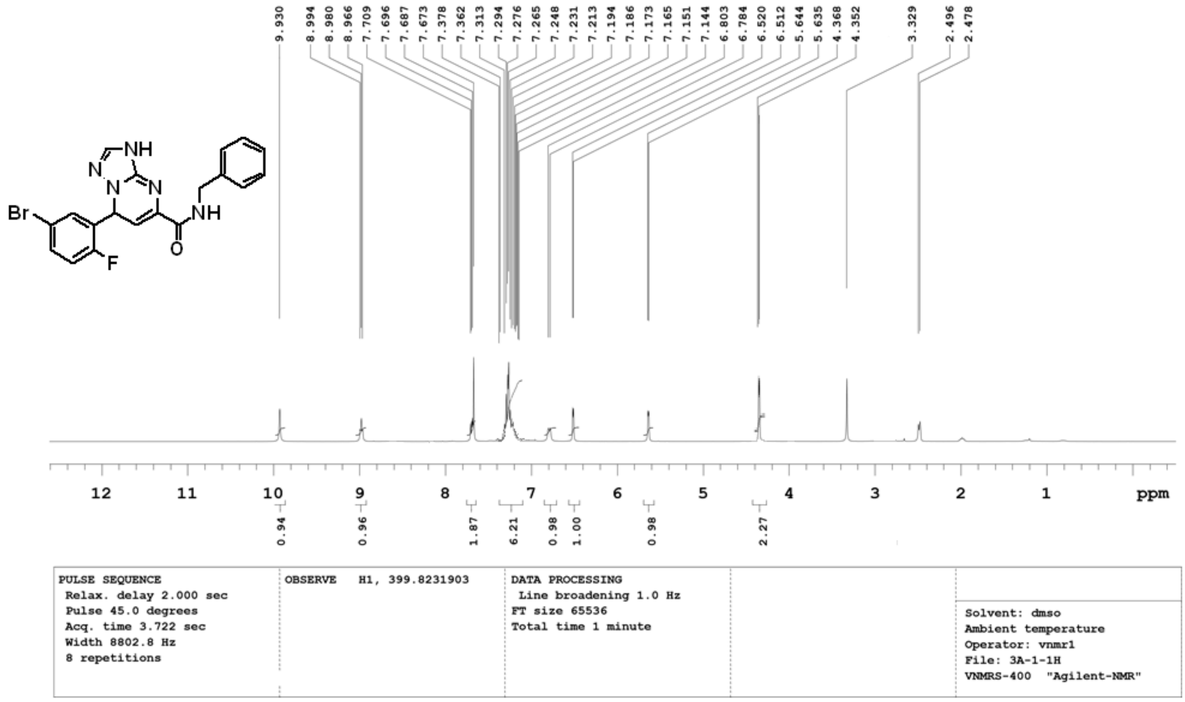

^{13}C NMR of Compound of **6ce**

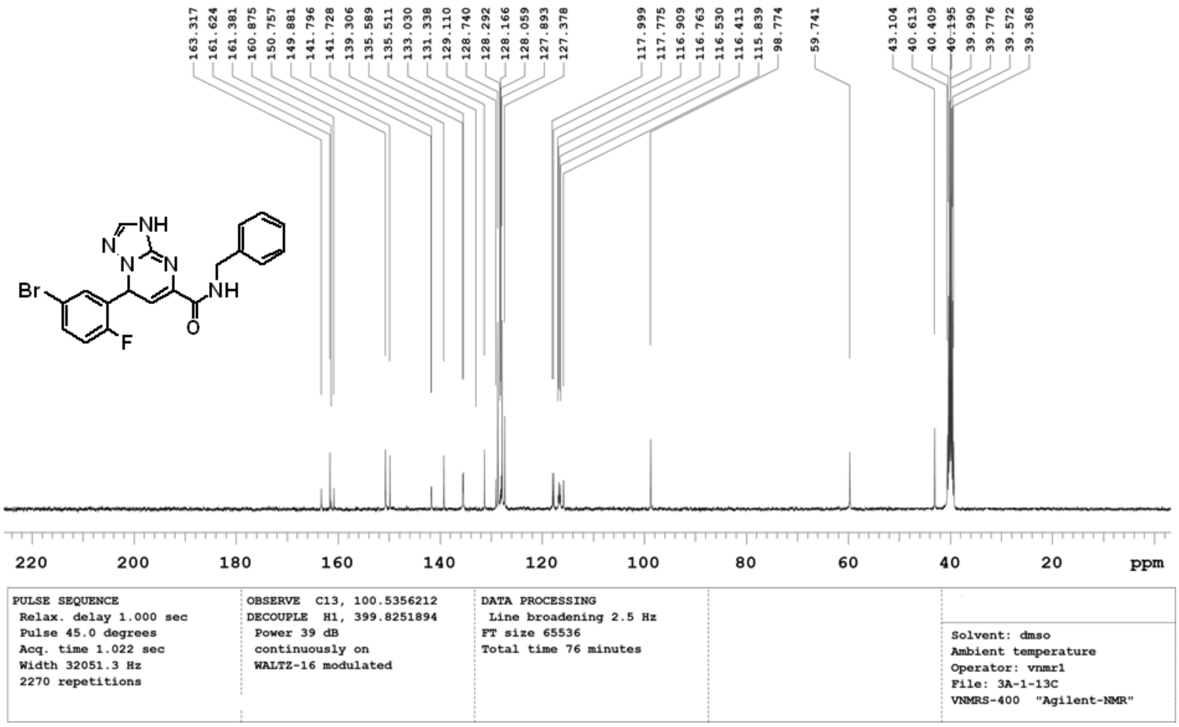

^{1}H NMR of Compound of **6cf**

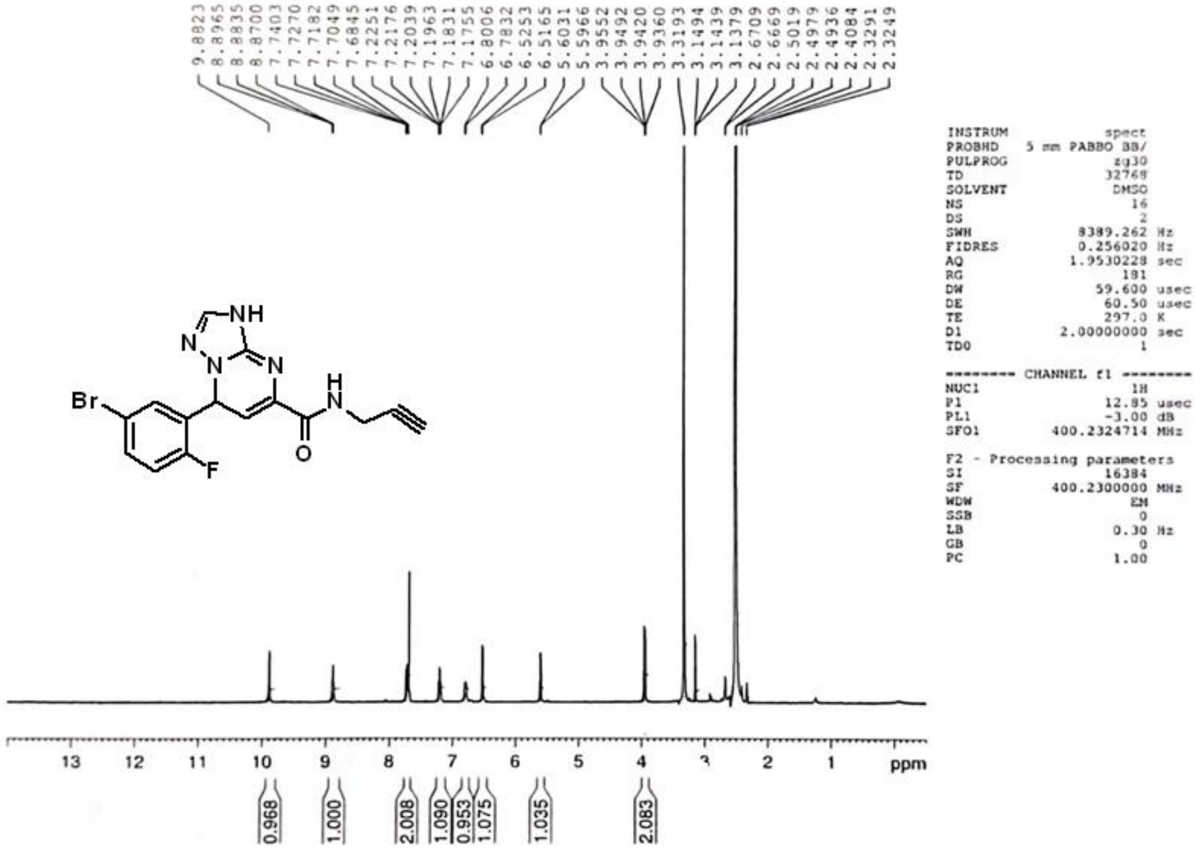

^{1}H NMR of Compound of **6cg**

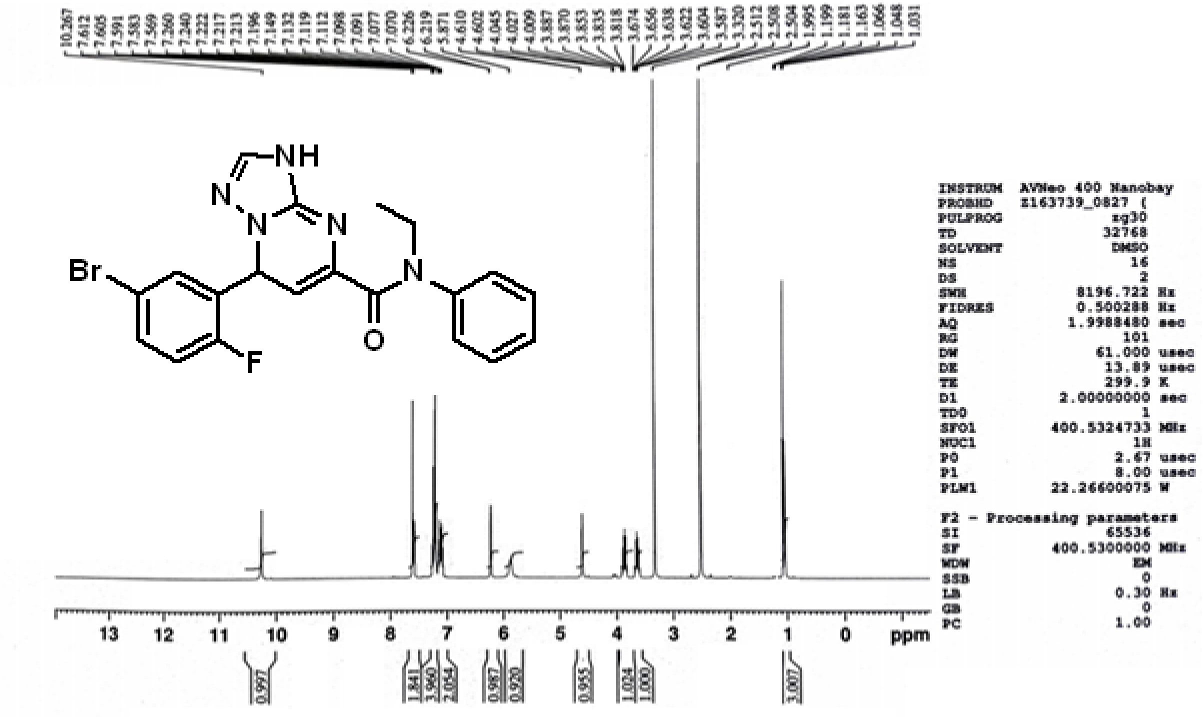

^{13}C NMR of Compound of **6cg**

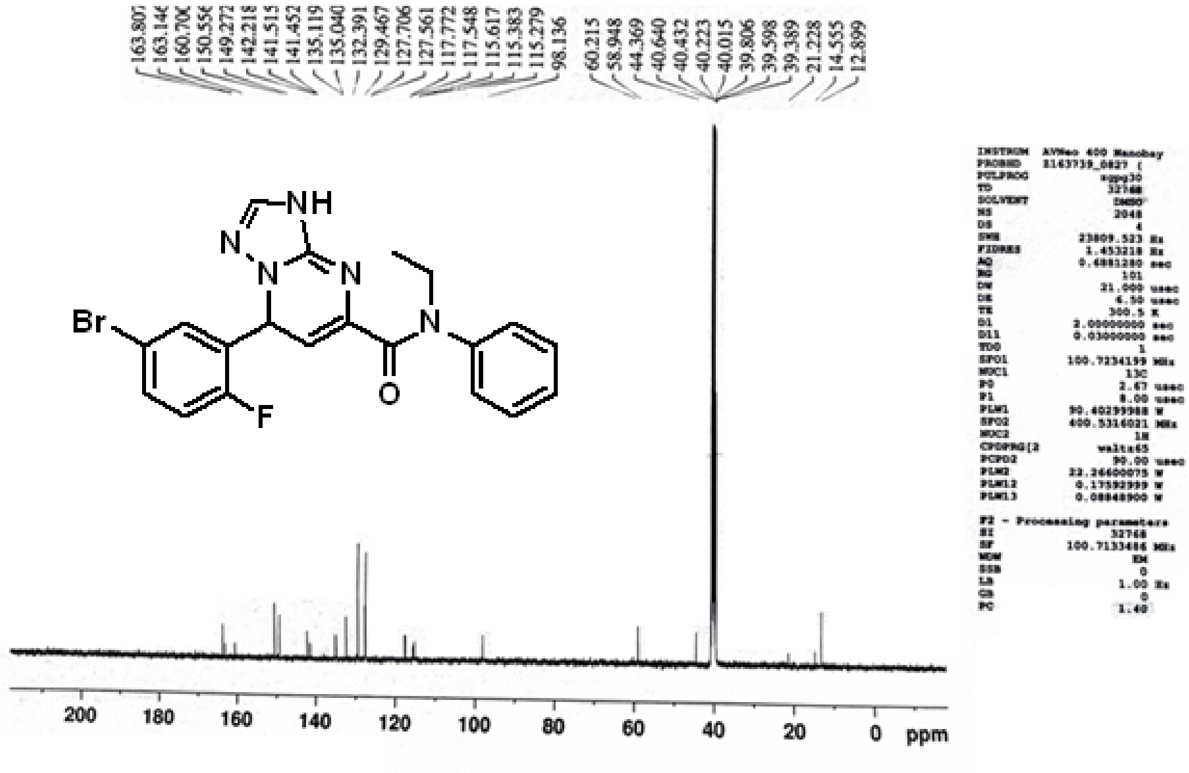

^{1}H NMR of Compound of **6ch**

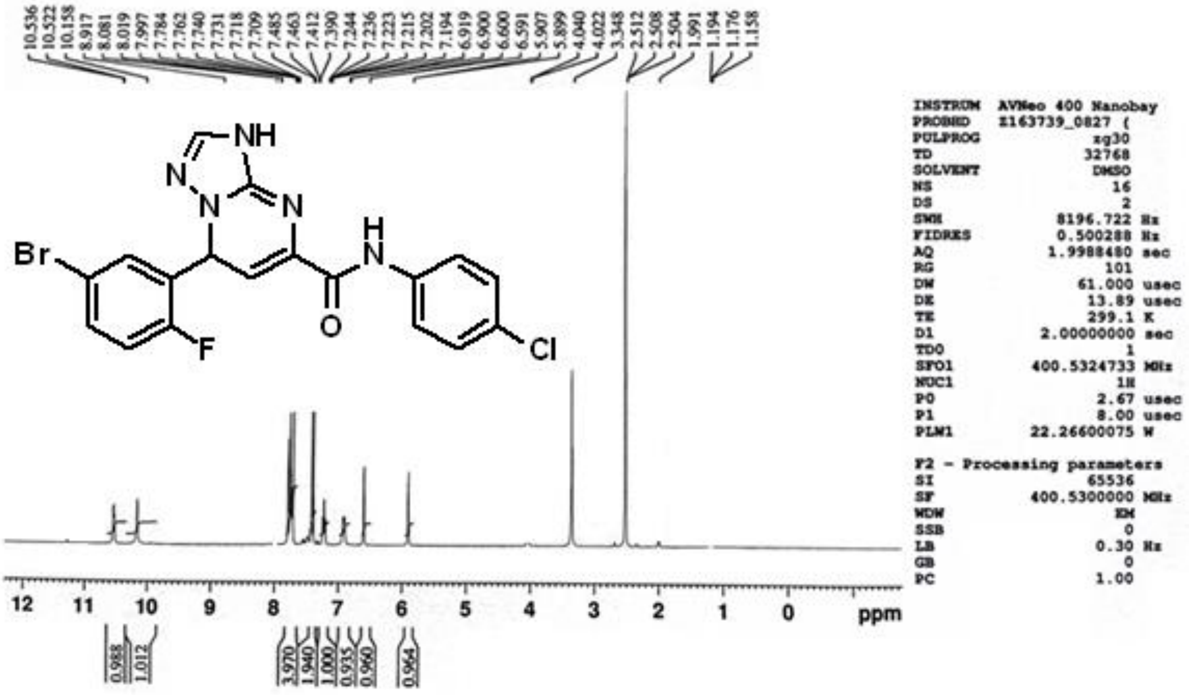

^{13}C NMR of Compound of **6ch**

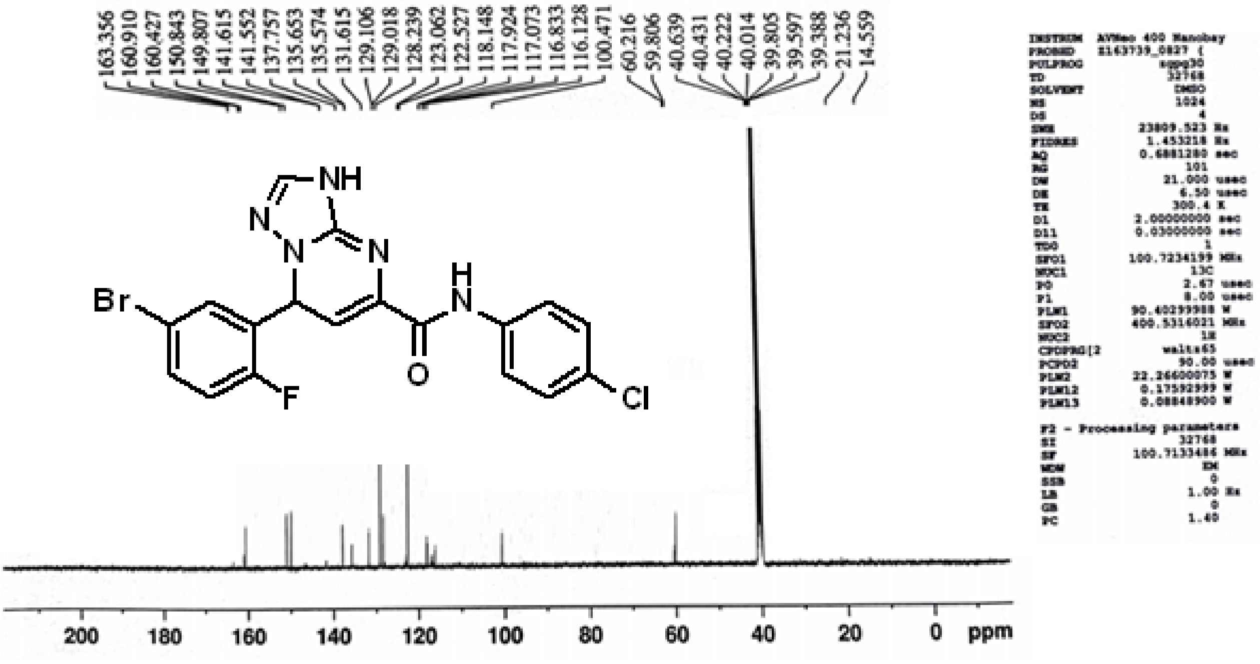

^{1}H NMR of Compound of **6ci**

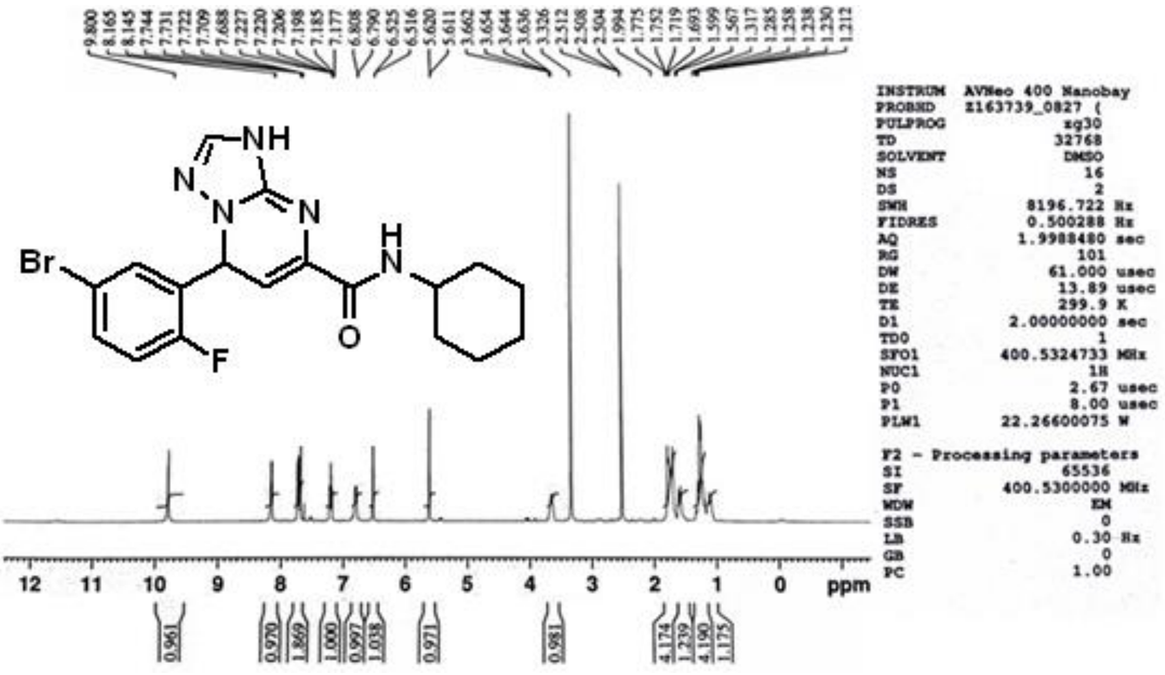

^{13}C NMR of Compound of **6ci**

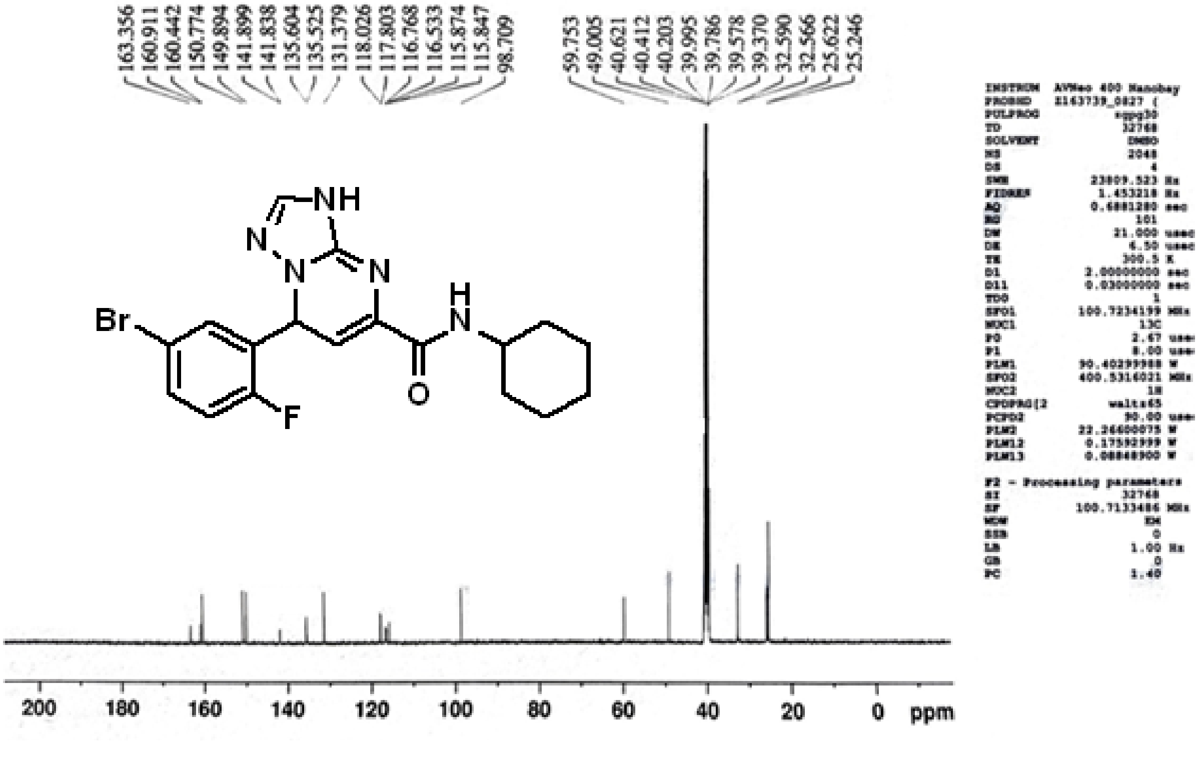

Supplement: Supplementary file 1 [file pharmaceuticals-17-00548-s001.zip › pharmaceuticals-2758234-supplementary.pdf]
